# Compact deep learning models for colon histopathology focusing performance and generalization challenges

**DOI:** 10.1038/s41598-026-35119-y

**Published:** 2026-01-19

**Authors:** Fareeha Hanif, Ali Raza, Heba Abdelgader Mohammed

**Affiliations:** 1 Department of Mathematics, University of Education, Vehari Campus, Lahore, Pakistan; 2 Department of Mathematics, University of the Punjab, Quaid e Azam Campus, Lahore, Pakistan; 3 Technical and Engineering Specialties Unit, Applied College, King Khalid University, Mohyel Asser, Kingdom of Saudi Arabia

**Keywords:** Colorectal cancer classification, Histopathological images, Lightweight CNN, Model comparison, Convolutional neural networks, Cancer, Computational biology and bioinformatics, Engineering, Mathematics and computing

## Abstract

Colorectal cancer is a leading cause of cancer-related mortality, and accurate analysis of histopathological images is critical for early diagnosis and improved patient outcomes. This study proposes and systematically evaluates four purpose-built lightweight convolutional neural network (CNN) variants (Lite-V0, Lite-V1, Lite-V2, and Lite-V4) for binary classification of colon histopathology images into *Colon_Adenocarcinoma* and *Colon_Benign_Tissue*. Experiments were conducted on a balanced dataset (24,000 images) with fixed train/validation/test splits and comprehensive evaluation using accuracy and macro-F1, supported by confusion matrices and ROC/precision–recall analyses. Among all variants, Lite-V2 achieved the best validation performance (macro-F1 $\approx$ 0.999), while remaining highly compact (1.53 MB; 127,682 parameters), indicating a favorable accuracy–efficiency trade-off for deployment-oriented diagnostic support. On the independent test set, however, Lite-V2 exhibited a marked generalization drop, achieving approximately 50% accuracy and macro-F1 = 0.33, suggesting a domain-shift effect between validation and test samples. These findings demonstrate that lightweight CNNs can achieve near-perfect internal validation performance for colon histopathology classification, but robust cross-domain generalization remains essential; future work will focus on domain adaptation and stain-robust training strategies to improve reliability on unseen clinical data.

## Introduction

This section introduces colorectal (colon) cancer as a major health burden, motivates the challenge of histopathological image interpretation, and positions convolutional neural networks (CNNs) as powerful tools to assist in diagnosis. Colon cancer remains a leading cause of cancer-related mortality worldwide, with histopathological examination serving as the diagnostic gold standard. Traditional diagnosis by pathologists is time-consuming and subject to inter-observer variability, creating a pressing need for automated classification systems.

### Background

Colorectal cancer (CRC) ranks among the most commonly diagnosed malignancies worldwide and remains a leading cause of cancer-related mortality. According to GLOBOCAN 2022 estimates, CRC accounted for approximately 10% of all new cancer cases and 9% of global cancer deaths^1^. In recent years, incidence rates have shown rising trends especially in younger populations, further intensifying the demand for efficient and accurate diagnostic systems^2^. As population screening and colonoscopic examinations become more widespread, pathology services face a growing caseload of tissue specimens requiring timely evaluation. The gold standard for definitive CRC diagnosis is histopathological examination of biopsies or resected specimens, typically via light microscopy of hematoxylin & eosin (H&E)–stained slides. Pathologists examine morphological features glandular structure, cellular atypia, architectural distortion, invasion to distinguish adenocarcinoma from benign tissue and to grade dysplasia. However, this manual workflow is labor-intensive, time-consuming, and subject to intra- and inter-observer variability, particularly in borderline or subtle cases. In many pathology departments, the rising volume of cases and limited human resources impose significant delays and workload pressures.

The digitization of pathology scanning glass slides into whole-slide images (WSIs) has paved the way for computational methods to assist pathologists. In this digital pathology realm, convolutional neural networks (CNNs) have demonstrated strong performance in classification, segmentation, and feature extraction tasks directly from image pixels. Studies have successfully applied CNN models to classify colorectal tissue patches into tumor or normal classes, predict prognostic indicators, or identify histologic subtypes. For example, Mohamed et al. developed a CNN combined with a Fisher Mantis optimizer for colon cancer diagnosis, achieving compelling accuracy on benchmark datasets^3^. Another work by Karthikeyan et al. presents a ranking-based CNN model tailored for colorectal cancer detection, showing robust results on independent test sets^5^. Beyond individual experiments, systematic reviews and meta-analyses have assessed the diagnostic accuracy of AI methods in pathology at large. McGenity et al. conducted a diagnostic-accuracy meta-analysis over 100 AI-based pathology studies, reporting a mean sensitivity of 96.3% and specificity of 93.3% across disease applications, while noting substantial heterogeneity and risk-of-bias in many studies. More broadly, reviews of computational pathology underscore that while AI systems show promise, challenges remain in generalizability, data diversity, and method transparency^6^.

Despite this promising progress, many high-performing CNN models are large, computationally intensive, and unsuitable for deployment in constrained environments or real-time pathology workflows. The gap between research prototypes and clinically deployable systems motivates the exploration of compact, lightweight CNN architectures that strike a balance between inference efficiency and diagnostic accuracy. In this work, we propose and investigate four lightweight CNN variants (Lite-V0, Lite-V1, Lite-V2, Lite-V4) designed for binary classification of *Colon_Adenocarcinoma* vs. *Colon_Benign_Tissue*. We also develop an end-to-end reproducible pipeline with augmentation, class weighting, macro-F1–based early stopping, and comprehensive evaluation to benchmark these architectures under realistic constraints.

### CNNs in medical imaging

Convolutional neural networks (CNNs) have revolutionized pattern recognition tasks in medical imaging by enabling end-to-end feature learning directly from raw pixels, thereby eliminating the need for hand-crafted feature engineering and facilitating robust hierarchical representations of texture, morphology, and contextual information in histopathological images. Early CNN architectures originally developed for natural image analysis, such as VGG and AlexNet, were rapidly adopted for medical imaging tasks, demonstrating that filters learned from large-scale datasets could be effectively transferred to capture diagnostically relevant tissue patterns. Subsequent architectural innovations, including residual learning in ResNet, alleviated vanishing-gradient issues and enabled deeper networks with improved feature reuse and stability during training. More recent families, such as EfficientNet, introduced compound scaling strategies to jointly optimize depth, width, and resolution, achieving strong accuracy with significantly fewer parameters and computational cost.

Beyond general-purpose CNNs, a substantial body of recent medical imaging research has focused on *lightweight and computationally efficient architectures* designed explicitly for deployment-oriented scenarios. Models such as MobileNet, ShuffleNet, NASNetMobile, and EfficientNet-Lite rely on depthwise separable convolutions, channel shuffling, or neural architecture search to reduce parameter count and inference latency while preserving discriminative power. These architectures have become common baselines in medical classification tasks, including brain tumor detection, diabetic retinopathy analysis, and histopathological cancer diagnosis, due to their favorable accuracy–efficiency trade-offs^25,28,29^. For example, comparative studies in brain tumor classification demonstrate that MobileNet and NASNetMobile achieve competitive accuracy relative to deeper CNNs while significantly reducing computational overhead^25,28^. Similarly, large-scale analyses in ocular disease and diabetic retinopathy detection highlight MobileNet’s robustness and efficiency across CNN and hybrid CNN–LSTM frameworks^29^.

The impact of CNNs in histopathology and broader medical imaging can therefore be summarized along several practical axes:

1. **Patch-level classification:** CNNs remain highly effective for patch-level prediction tasks, where regions extracted from whole-slide images (WSIs) are classified into diagnostic categories such as benign versus malignant tissue or cancer subtypes. Lightweight CNNs trained either from scratch or with limited pretraining have shown excellent performance in colon, lung, breast, and skin cancer histopathology, achieving accuracies above 95% while maintaining compact model sizes^11,23,31,32^.
2. **Transfer learning vs. from-scratch training:** Transfer learning using ImageNet-pretrained backbones (e.g., ResNet, MobileNet, EfficientNet) is widely adopted to improve convergence speed and generalization when labeled medical data are limited. However, recent evidence suggests that carefully designed lightweight CNNs trained from scratch can achieve comparable or superior performance with reduced architectural complexity and domain mismatch, particularly in histopathology settings^23,27^.
3. **Model efficiency and deployment:** Architectures such as MobileNet, ShuffleNet, EfficientNet-Lite, and NASNetMobile explicitly target low-parameter regimes and fast inference, making them suitable for deployment in clinical environments, low-resource laboratories, or edge devices. Comparative evaluations consistently demonstrate that these lightweight models strike an effective balance between accuracy, memory footprint, and inference cost^24,25,29^.
4. **Hybrid and attention-enhanced CNNs:** To overcome the representational limitations of lightweight backbones, many studies integrate attention mechanisms, multi-scale feature fusion, or pyramidal structures on top of efficient CNNs. Such hybrid designs improve localization accuracy and robustness to staining or scanner variability, as demonstrated in lung, brain, prostate, and skin cancer studies^11,28,33,34^.
5. **Explainability and clinical validation:** Gradient-based visualization techniques, such as Grad-CAM, are commonly applied to both heavyweight and lightweight CNNs to provide visual explanations that support clinical interpretability. Recent works emphasize that explainability, external validation, and computational efficiency must be jointly addressed to ensure reliable real-world deployment of CNN-based diagnostic systems^11,31,35^.

Building on these computational trends, the present study focuses on a systematic comparison of purpose-built lightweight CNN variants (Lite-V0, Lite-V1, Lite-V2, Lite-V3) trained end-to-end for colon histopathology classification. By emphasizing reproducible training pipelines, macro-F1-based evaluation, and explicit accuracy–complexity trade-offs, our work directly addresses the practical limitations identified in existing MobileNet-, ShuffleNet-, and EfficientNet-based approaches, while targeting clinically relevant deployment constraints.

### Motivation and challenges

Designing effective deep learning models for histopathological image classification, especially in resource-constrained or clinical settings, involves confronting multiple interlocking challenges. In this subsection, we articulate four key challenge dimensions that motivate the development of lightweight CNN variants.

#### Model complexity and size

Many state-of-the-art CNN architectures (e.g., VGG, ResNet, DenseNet) consist of tens to hundreds of millions of parameters, resulting in large memory footprints and high storage demands. Their large size often hinders deployment to edge devices or clinical workstations with limited hardware resources. For instance, VGG-16 alone has around 138 million parameters and a model file size exceeding 500 MB^14^. In histopathology, researchers have proposed compact models like ReducedFireNet, achieving competitive accuracies while maintaining model sizes under 0.5 MB^15^. Similarly, recent extremely lightweight CNN designs (e.g. ELW-CNN) have been demonstrated on lung and colon image datasets with high accuracy and minimal size^51^.

#### Computational cost and inference efficiency

Large, deep CNNs demand substantial computational resources for both training and inference (e.g., high FLOPs, GPU memory, and energy). In many medical settings, real-time or near-real-time inference is desired, which large networks struggle to achieve. Moreover, limited hardware (e.g., CPUs or lower-end GPUs) cannot always support heavy architectures. Lightweight CNNs reduce FLOPs and computational load, making inference faster and more practical for on-site diagnostics^17^. Studies in medical image classification indicate that reducing computational cost is critical for adoption in low-resource environments^18^.

#### Limited annotated data and overfitting risks

Deep networks typically require large datasets to generalize well. In medical imaging and especially histopathology–annotated datasets are often small, limited by expert labeling cost and privacy constraints. This paucity of training data increases the risk of overfitting and domain-specific bias. Lightweight architectures, with fewer parameters, help mitigate overfitting risks by reducing model capacity relative to data size. Several studies advocate designing small but expressive networks for medical domains as a buffer against overfitting^18,19^.

#### Trade-off between compactness and performance

A central tension is achieving strong predictive performance while keeping model complexity low. Too lightweight a model may underfit and lose crucial discriminative power; too large a model may overfit or be impractical. The ideal balance lies in architectures that deliver near state-of-the-art accuracy with significantly reduced parameters and computational cost. This trade-off motivates systematic comparison of lightweight variants (Lite-V0, Lite-V1, Lite-V2, Lite-V4) to identify architectures that achieve optimal balance in the colon cancer histopathology domain. Together, these challenge dimensions justify our focus on designing and evaluating lightweight CNN variants tailored to histopathological classification tasks. In subsequent sections, we describe how our proposed models, training pipeline, and evaluation strategy address these challenges in a reproducible and performance-sensitive manner.

### Study objectives and contributions

This subsection lists the concrete objectives of the present study and explains the main contributions. The goals are stated concisely as a bullet list and then each goal is expanded in a short subsubsection. The design is motivated by recent work on lightweight CNNs, transfer learning, augmentation strategies, and reproducible evaluation pipelines in histopathology^20,44,86^.

1. Develop four lightweight CNN variants (Lite-V0 to Lite-V4) We design and implement four progressively complex CNN variants Lite-V0, Lite-V1, Lite-V2, and Lite-V4 that incrementally increase filter counts and depth while preserving a compact overall parameter footprint. The purpose is to systematically explore the parameter–performance frontier so that the final recommendation balances predictive performance with model size and inference efficiency, an approach aligned with recent lightweight and multiscale CNN work in colon/lung histopathology^21,44^.
2. Design a robust training pipeline with augmentation and F1-based early stopping We construct an end-to-end training pipeline incorporating standard preprocessing (224$\times$224 resizing and normalization), extensive data augmentation (rotations, flips, scaling, color jitter where appropriate), balanced class handling via computed class weights, and early stopping driven by validation macro-F1. Using macro-F1 as the stopping criterion emphasizes balanced performance across classes and is recommended in medical imaging contexts where per-class errors carry different clinical costs^22,86^.
3. Evaluate and compare models using accuracy, loss, F1, and ROC/PR metrics Model evaluation employs a suite of quantitative metrics: accuracy and loss for global behavior, macro-F1 for class-balanced performance, confusion matrices for per-class error analysis, and ROC / precision–recall curves for threshold-independent assessment. We follow best-practice recommendations for multi-metric reporting and visual diagnostics widely discussed in recent computational pathology literature^20^.
4. Identify the best-performing model for colon cancer classification Using the validation macro-F1 as the model-selection criterion, we identify the top-performing variant and then evaluate it on an independent test set, reporting final test accuracy, macro-F1, confusion matrix, ROC/PR curves, and a CSV of per-image predictions (including filepath, true label, predicted label, confidence, and correctness). All artifacts (saved best model files, curves, confusion matrices, ROC/PR images, and prediction CSV) are produced to ensure reproducibility and easy downstream analysis^44^.
  **Novelty statement:** “This study proposes an efficient CNN-based framework with systematic comparison for colon cancer diagnosis.”

The multi-class output is operationally more informative and robust for several reasons:

- Develop four lightweight CNN variants (Lite-V0 to Lite-V4).
- Design a robust training pipeline with augmentation and macro-F1–based early stopping.
- Evaluate and compare models using accuracy, loss, macro-F1, and ROC/PR metrics.
- Identify the best-performing model for colon cancer classification and report reproducible artifacts.

## Expanded and categorized review of CNN-based medical image classification

Recent advances in deep learning have significantly transformed medical image analysis, particularly in cancer diagnosis, neurological disorders, and pathological tissue classification. While earlier studies primarily focused on improving classification accuracy, recent research has shifted toward lightweight architectures, explainable artificial intelligence (XAI), and clinically deployable frameworks. However, the existing literature remains fragmented across different classification paradigms, including patch-level versus whole-slide analysis, transfer learning versus from-scratch training, and heavyweight versus lightweight CNN models. This section categorizes and critically reviews prior studies to clearly position the contribution of the present work.

### Patch-level vs. whole-slide image classification

Patch-level classification remains the dominant paradigm in histopathological image analysis due to memory and computational constraints. Li *et al.* ^23^ proposed a lightweight CNN trained from scratch for patch-level colon cancer tissue classification, achieving a test accuracy of $0.990 \pm 0.003$ while maintaining a compact model size. Similarly, Banerjee ^11^ introduced the DY-FSPAN framework for lung cancer histopathology, leveraging pyramidal attention mechanisms to enhance patch-level feature representation and interpretability. Patch-based strategies have also been extensively applied in breast ^32^, skin ^31^, and prostate cancer ^33^ analysis, where fine-grained tissue discrimination is essential.

In contrast, whole-slide image (WSI) analysis remains underexplored due to its computational complexity and annotation challenges. Most existing works either avoid full-slide analysis or rely on weakly supervised aggregation of patch-level predictions, highlighting a clear research gap for efficient and scalable WSI-level solutions.

### Transfer learning vs. from-scratch learning

Transfer learning has been widely adopted to compensate for limited medical datasets. Ke *et al.*^27^ employed domain-specific transfer learning with multi-model feature fusion and attention mechanisms, achieving accuracies exceeding 99% on multiple colorectal cancer datasets. Comparative studies on brain tumor detection^25,28,34^ consistently show that pre-trained architectures such as VGG, MobileNet, NASNet, and ResNet provide strong baseline performance when combined with fine-tuning strategies.

However, reliance on pre-trained natural image features introduces domain mismatch and increases model complexity. From-scratch lightweight models, such as the one proposed in^23^, demonstrate that carefully designed CNNs can achieve competitive or superior performance while reducing parameter count, memory footprint, and inference cost. This distinction is particularly critical for deployment in resource-constrained clinical environments.

### Lightweight and attention-based architectures

Lightweight CNNs and attention-driven frameworks have emerged as a key research direction. MobileNet-, ShuffleNet-, and NAS-based architectures are frequently used as backbones due to their efficiency^25,29^. More recent works integrate attention mechanisms to enhance interpretability and localization, such as pyramidal attention^11,28^, kernel attention^16^, and hybrid convolution-attention models^30,33^. These methods improve class-wise discrimination and reduce false positives, but often at the expense of architectural complexity and increased training overhead.

### Explainability, multi-modality, and review studies

Explainable AI has become a critical requirement for clinical trust. Several studies employ Grad-CAM and attention visualization to highlight diagnostically relevant regions^11,28,31,34^. Multi-modal fusion strategies are reviewed extensively in^26^, demonstrating improved robustness for brain disease detection. Comprehensive review articles further summarize progress and challenges across diabetic retinopathy^24^, skin cancer^31^, breast cancer^32^, and cardiovascular disease prediction^35^, consistently emphasizing the lack of external validation, model transparency, and computational efficiency as unresolved challenges.

### Comparative summary of existing literature

Table 1 summarizes representative studies, categorizing them by classification approach, learning strategy, dataset, performance, and key limitations.

**Table 1:** Comparative summary of CNN-based medical image classification studies.

**Reference**	**Task**	**Approach**	**Learning Strategy**	**Performance**	**Limitations**
^23^	Colon cancer	Patch-level CNN	From-scratch, lightweight	Acc. 99.0%	Limited WSI analysis
^25^	Brain tumor	CNN comparison	Transfer learning	Acc. 98.79%	Heavy architectures
^27^	Colorectal cancer	Feature fusion + attention	Transfer learning	Acc. 99.68%	High complexity
^11^	Lung cancer	Pyramidal attention CNN	From-scratch	Acc. 98.5%	Computational overhead
^28^	Brain tumor	Attention-based hybrid	Transfer learning	Acc. 99.12%	Large model size
^31^	Skin cancer	Explainable CNN	Hybrid attention	Acc. 95.92%	Dataset dependency
^32^	Breast cancer	Residual attention CNN	Transfer learning	Acc. 99.23%	Limited external validation
^33^	Prostate cancer	Multi-block segmentation	Attention-based	Dice 0.986	High training cost
^34^	Brain tumor	FSPAN-based CNN	Lightweight hybrid	Acc. 99.89%	Multi-dataset fusion needed
^35^	Heart disease	ML/DL review	Various	Acc. up to 99%	Clinical translation gap

Overall, the literature reveals a clear trend toward lightweight, interpretable, and high-performing CNN architectures. Nevertheless, challenges remain in balancing model efficiency, explainability, and generalization. These gaps motivate the development of optimized lightweight CNN frameworks that can achieve robust performance without reliance on heavy pre-trained models or excessive computational resources.

## Related work

### CNN-based colon cancer classification

Convolutional neural networks (CNNs) have become pivotal in histopathological image analysis of colon and gastrointestinal tissues, enabling direct learning of discriminative features from raw image patches. These models have been widely used to distinguish malignant from benign tissue, classify subtypes, and assist in region-of-interest detection. Below we organize recent work into four thematic sub-subsections.

#### Patch-level classification on benchmark datasets

A foundational line of research uses CNNs to classify small patches cropped from whole-slide images (WSIs) on public datasets such as LC25000 and Kather CRC. Several studies have trained lightweight CNNs or fine-tuned deeper networks to achieve pointwise classification accuracy often exceeding 95%^36–38^. While these results are promising, they frequently rely on within-dataset validation, limiting insight into cross-institutional robustness.

#### Transfer learning and fine-tuning strategies

To overcome limited annotated data, many works adopt transfer learning: initializing models with weights pretrained on ImageNet (e.g. VGG, ResNet, EfficientNet) and then fine-tuning on histopathology images. This strategy accelerates convergence and enhances generalization, and has been successfully applied in colorectal cancer tasks with high accuracy^39^. Nonetheless, the resulting models may still be computationally heavy for deployment.

#### Inference-time efficiency and lightweight architectures

The computational footprint of large CNNs hampers deployment in clinical settings or on resource-limited hardware. To mitigate this, a subset of research explores compact network architectures (e.g. MobileNet variants, shallow custom CNNs) or model compression techniques to reduce inference time and memory usage^37,40^. These methods typically trade a minor drop in accuracy for substantial gains in speed and resource efficiency.

#### Explainability, generalization, and external validation

- Explainability for Clinical Applicability: Many recent studies incorporate explainability methods (e.g., Grad-CAM, saliency maps) to visualize decision regions and enable interpretation by pathologists.
- Challenges in Generalization: External validation on independent datasets has revealed significant domain shift issues, where models performing excellently on internal validation often degrade on unseen data^38^.
- Need for Rigorous Reporting: Systematic reviews emphasize the necessity of reporting full diagnostics (e.g., confusion matrices, ROC/PR curves, calibration), not just accuracy, to properly assess real-world viability^42,52^.
- Overall Assessment: CNN-based approaches demonstrate strong potential for patch-level classification and transfer learning in colon histopathology.
- Remaining Challenges: Key challenges of inference cost, generalizability, and clinical explainability motivate the pursuit of lightweight CNN variants within a rigorous, reproducible framework.

### Lightweight and custom CNN approaches

The deployment of convolutional neural networks (CNNs) in clinical or resource-constrained settings requires careful balancing of model size, inference speed, and diagnostic accuracy. In histopathology, especially for colon tissue classification, researchers have increasingly turned to lightweight and custom-designed CNNs to meet these constraints. Below, we review several approaches categorized into five themes, illustrating how small models are adapted or invented for medical imaging tasks.

#### MobileNet and derivatives in medical imaging

MobileNet architectures (V1, V2, V3) are frequently used as base models for pathology tasks due to their use of depthwise separable convolutions, inverted residual blocks, and low compute demands. These features make them well-suited for patch-level classification on whole-slide images with limited computational resources. In colorectal histopathology, hybrid versions of MobileNet or “Mobile-EfficientNet” have been fine-tuned with good success, providing a strong baseline for lightweight medical models^43,44^. Their modular structure also allows for pruning and quantization, further reducing latency.

#### ShuffleNet and group-convolution based networks

ShuffleNet (V1/V2) achieves efficiency via grouped pointwise convolutions combined with channel-shuffle operations to mix information across groups. This design minimizes computation while retaining expressive power. In histopathology and related biomedical imaging, lightweight versions of ShuffleNet have been explored, often in ensemble with other models, to reduce inference cost while preserving accuracy^45–47^. These architectures provide a viable path when hardware imposes stringent limits on memory or GPU compute.

#### EfficientNet, EfficientNet-Lite, and compound scaling

EfficientNet introduced the strategy of compound scaling simultaneously scaling width, depth, and input resolution–to maximize accuracy relative to parameter count. Its Lite variants (e.g. EfficientNet-Lite or Mobile-EfficientNet) further reduce parameters and latency, making them attractive for medical image tasks. In colon cancer image classification and other histopathology applications, researchers have fine-tuned EfficientNet/Lite backbones for strong performance with manageable compute overhead^48,50^. Recently, Ochoa-Ornelas et al. used EfficientNetB3 in a transfer learning ensemble for colon and lung cancer detection, emphasizing improved detection with moderate resource demands^50^.

#### Extremely lightweight architectures and domain-specific designs

- Lightweight CNN: A non-pretrained architecture with approximately 4.4 million parameters that achieved  99.0% test accuracy on colon histopathology datasets, showcasing the effectiveness of domain-specific optimization .
- Integrated Design Approach: These specialized designs typically combine data cleaning procedures, architecture simplification strategies, and built-in visualization capabilities (e.g., Grad-CAM) specifically tailored for clinical deployment constraints.
- Performance-Parameter Efficiency: The core objective remains achieving maximal classification performance while minimizing the parameter count and computational requirements for practical clinical implementation.

#### Preference and constraints for real-time clinical use

In clinical environments, smaller models are preferred for several pragmatic reasons. Biomedical AI reviews emphasize that deployment-ready systems must not sacrifice robustness or generalizability for compactness^53,54^. In practice, many clinical AI prototypes use lightweight CNN architectures or compressed versions of standard models to meet these constraints.

1. **Inference latency:** Faster execution enables real-time feedback during diagnostic review, crucial for time-sensitive clinical decisions.
2. **Memory and storage constraints:** Pathology workstations and edge devices typically have limited RAM, GPU memory, and disk space, necessitating efficient models.
3. **Energy consumption:** Lower compute demands reduce power usage and operational costs, supporting sustainable healthcare operations.
4. **Validation and transparency:** Simpler models are more amenable to interpretation via explanation maps and regulatory certification processes.

Together, these approaches illustrate the growing maturity of lightweight CNN design in medical imaging. Our work builds on this foundation by proposing and systematically comparing multiple lightweight CNN variants (Lite-V0, Lite-V1, Lite-V2, Lite-V4) specifically for colon histopathology classification.

### Research gap

Although deep learning has revolutionized digital pathology, several challenges and research gaps persist in the context of colon cancer histopathological image classification. A large number of recent studies have successfully demonstrated high diagnostic accuracy using convolutional neural networks (CNNs) for colorectal tissue classification; however, most of these approaches rely on large, pre-trained architectures that are computationally heavy and require substantial hardware resources for both training and inference^37,38^. These models, while powerful, are often unsuitable for integration into real-time diagnostic pipelines or low-resource clinical environments, where efficiency and speed are crucial. Moreover, many studies in this domain focus on evaluating a single deep network rather than systematically comparing multiple custom lightweight architectures trained under identical experimental conditions. This lack of standardized comparative studies leads to fragmented findings and makes it difficult to assess which architectural configurations best balance performance and computational efficiency^2,52^. Similarly, several published works report high in-sample accuracy but lack extensive cross-validation or testing on independent datasets, limiting generalizability and reproducibility across medical centers.

Another major limitation in the existing literature is the limited emphasis on model transparency and reproducible workflows. While some studies report performance metrics such as accuracy and F1-score, few provide comprehensive performance artifacts including confusion matrices, ROC and precision-recall (PR) curves, or per-image prediction logs. These outputs are critical for in-depth error analysis and for validating whether a trained model can be trusted for clinical support^50,51^. Additionally, the scarcity of open-access, standardized colon histopathology datasets further constrains the ability to compare results fairly and contributes to data imbalance and overfitting risks. Finally, only a handful of studies have specifically optimized lightweight CNNs for the morphological patterns unique to colon tissues, such as glandular architecture, texture variations, and staining inconsistencies^55,56^. Many lightweight CNNs are adapted directly from natural image recognition tasks, without sufficient domain adaptation or exploration of how network depth, filter scaling, and pooling strategies influence colon cancer classification accuracy. To address these gaps, the present study introduces a reproducible and transparent framework for colon cancer classification using four lightweight CNN variants (Lite-V0, Lite-V1, Lite-V2, and Lite-V4). Each model is trained from scratch using a standardized data augmentation, class-weight balancing, and F1-based early stopping strategy. The proposed pipeline generates comprehensive artifacts–including accuracy/loss/F1 training curves, ROC and PR plots, confusion matrices, and per-image predictions–to enable transparent and fair comparison. Through this controlled evaluation, the study aims to establish an empirical foundation for designing efficient yet accurate lightweight CNNs suitable for real-world clinical applications in histopathology.

## Materials and methods

### Dataset description

The dataset used in this study is a two-class colon histopathology image collection designed to differentiate between *Colon_Adenocarcinoma* and *Colon_Benign_Tissue*. These two tissue categories represent malignant and normal colon structures, respectively, and are central to the early detection and diagnosis of colorectal cancer. Colon adenocarcinoma is characterized by the presence of irregular glandular architecture, nuclear atypia, and stromal invasion, whereas benign colon tissue exhibits uniform glandular patterns and well-organized epithelial cells. Correctly distinguishing these histological patterns is critical for pathologists to prevent false positives or missed cancer diagnoses^7,57^. The dataset structure follows a well-defined split to facilitate training, validation, and unbiased testing. It comprises approximately 21,000 images in the training set, 1,000 images for validation, and 2,000 images for testing, totaling 24,000 colon tissue images. Each split contains an equal representation of the two classes to ensure balanced learning and evaluation. The balance across training and testing stages minimizes bias toward any single class and allows performance metrics such as accuracy, F1-score, and AUC to reflect the model’s true discriminative capability. To maintain fairness during optimization, class weights were computed dynamically from the training data using the Scikit-learn utility function; however, since the dataset was nearly balanced, the computed weights for both classes were approximately equal (close to 1.0). These computed weights were still applied during training to account for potential subtle class distribution variations as Table 2.

This image collection is derived from the publicly available LC25000 dataset, introduced by Borkowski *et al.* in 2019^57^. The LC25000 dataset contains a total of 25,000 high-resolution histopathological images of lung and colon tissues across five classes. Out of these, two classes correspond to colon tissue–*Colon_Adenocarcinoma* and *Colon_Benign_Tissue*. Each class originally contained 5000 samples, which were synthetically expanded through controlled data augmentation techniques to form 25,000 total images. The original dataset was curated using histopathological slides obtained from multiple patients and digitized under consistent staining and imaging conditions. Subsequent preprocessing ensured uniform dimensions, lighting normalization, and contrast consistency. LC25000 and its colon-specific subsets have become standard benchmarks for evaluating deep learning models in gastrointestinal cancer research. All images in our study were processed in RGB format and resized to $224 \times 224$ pixels to align with CNN input specifications. Each pixel intensity was normalized to the $[0,1]$ range by dividing by 255. To promote robustness against overfitting, we applied real-time data augmentation during the training phase. The augmentations included random horizontal and vertical flips, small rotations (up to $\pm 15^{\circ }$), width and height shifts, and zoom variations within 10–20%. These transformations simulate histopathological variability due to differences in tissue sectioning, orientation, and staining. During validation and testing, images were not augmented to ensure unbiased evaluation. This standardized preprocessing and augmentation pipeline enhances generalization and aligns with prior studies that have employed similar strategies for histopathological image analysis^7,38,58^.

**Fig. 1:**
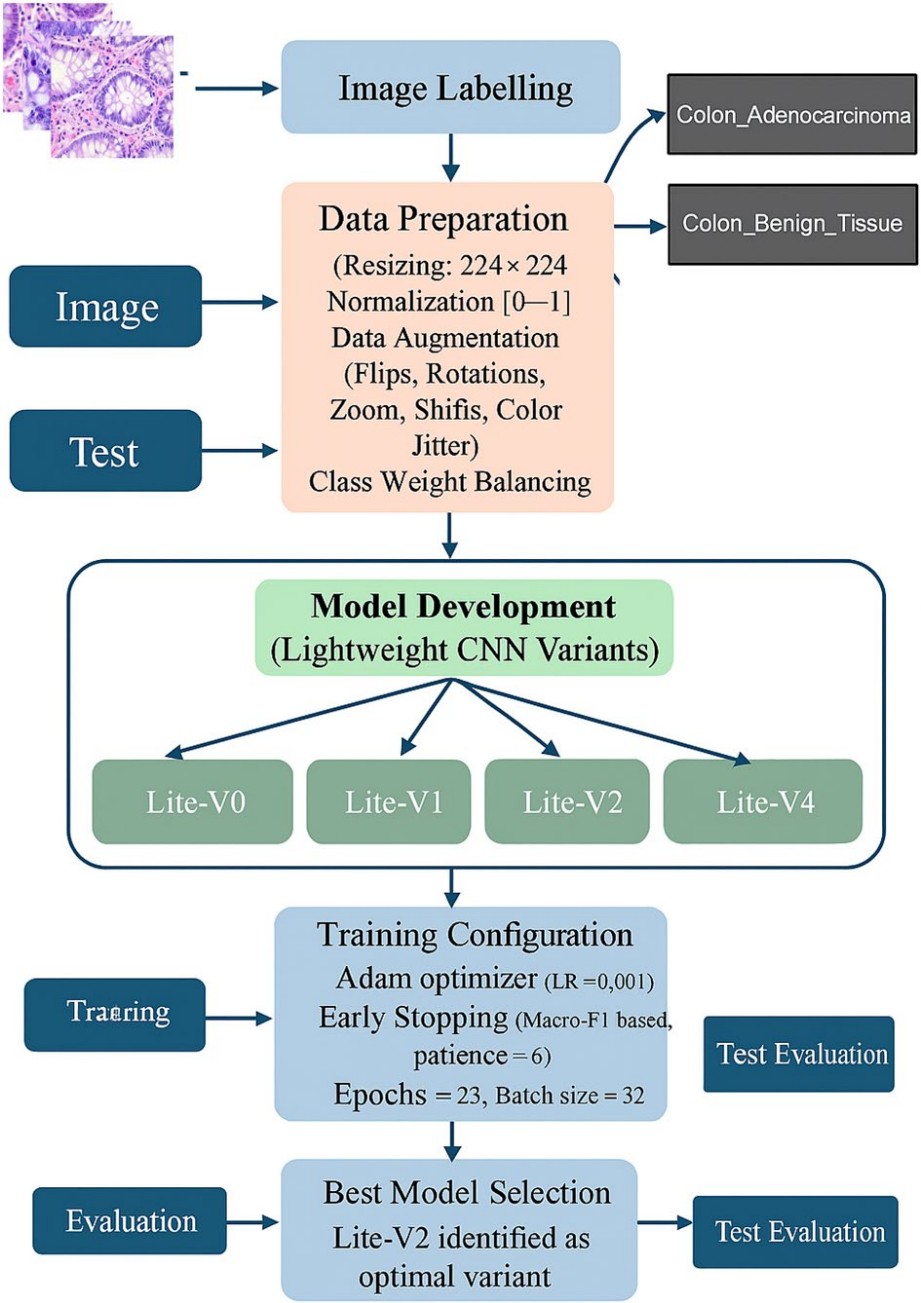
Methodology Based on Proposed Custom Convolutional Neural Network.

The dataset directory is hierarchically organized with three main folders: *train*, *val*, and *test*. Each folder contains two subfolders named according to the two classes–*Colon_Adenocarcinoma* and *Colon_Benign_Tissue*. During training, data loading and preprocessing were performed using the tf.keras.preprocessing.image_dataset_from_directory() function, which automatically assigns labels based on folder names and ensures consistent shuffling across epochs. The datasets were prefetched and batched using TensorFlow’s AUTOTUNE feature for optimal GPU utilization. The dataset is widely recognized in digital pathology research as an ideal benchmark for validating deep CNN architectures, particularly for studies emphasizing lightweight and efficient models. Many researchers have leveraged LC25000 or its subsets to evaluate transfer learning using VGG16, ResNet50, DenseNet121, MobileNetV2, and EfficientNet^2,56^. However, the majority of these works rely on large-scale pre-trained models that, although accurate, are computationally intensive. In contrast, our study focuses exclusively on the colon component of LC25000 and proposes a systematic comparison of lightweight CNN variants (Lite-V0, Lite-V1, Lite-V2, Lite-V4) trained from scratch on the same data splits as presented in Fig. 1. This design ensures that the performance differences observed are attributable solely to architectural changes rather than pretrained feature transfer or dataset partitioning inconsistencies. In summary, the dataset serves as a robust and well-curated foundation for evaluating colon cancer classification models. Its balanced structure, controlled augmentation, and standardized preprocessing make it suitable for benchmarking lightweight deep learning frameworks aimed at achieving efficient, high-accuracy diagnosis in histopathology.

**Table 2:** Statistical summary of the dataset, detailing sample distribution per class and across different data partitions.

Class Name	Train	Validation	Test	Total
Colon_Adenocarcinoma	10,500	500	1000	12,000
Colon_Benign_Tissue	10,500	500	1000	12,000
Total Images	21,000	1000	2000	24,000

### Data preparation

Robust data preparation is essential for training reliable convolutional neural networks on histopathological images, where variability in tissue preparation, staining protocols, and scanning conditions can introduce substantial appearance heterogeneity. In this work, all images were converted to RGB format (when required) and resized to $224 \times 224$ pixels to ensure compatibility with the convolutional backbones and to allow fair comparison across all lightweight model variants. This spatial resolution represents a commonly adopted trade-off between preserving diagnostically relevant morphological detail and maintaining computational efficiency in contemporary computational pathology pipelines^59,60^. Following resizing, pixel intensities were normalized to the $[0,1]$ range via min–max scaling (division by 255). Because the models were trained from scratch rather than initialized with ImageNet-pretrained weights, this normalization strategy avoids introducing mismatched channel statistics that may arise when applying ImageNet mean–standard deviation normalization in non-natural image domains^59,61^.

**Fig. 2:**
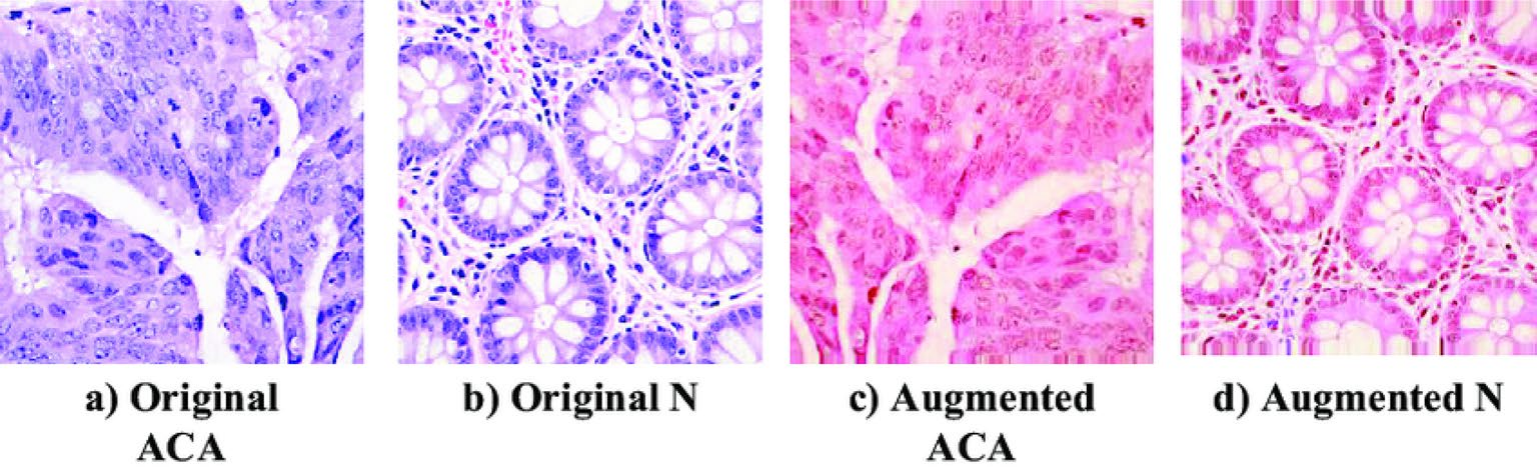
Example of Augmented Colon Histopathological Images to illustrate variations.

*Stain variability, noise, and artifact handling.* Histopathological images are affected by staining variability (particularly in H&E slides), scanner-dependent color responses, and acquisition noise, some samples are shown in Fig. 2. To address these factors, we adopted a conservative preprocessing strategy. First, explicit denoising filters (e.g., Gaussian or median filtering) were not applied by default, as excessive smoothing may suppress diagnostically meaningful cellular boundaries and texture patterns. Instead, robustness to mild noise was achieved implicitly through data augmentation and model regularization, consistent with recent findings that CNNs can learn noise-invariant representations when trained with sufficient variability^68,86^. Second, stain variability was addressed primarily through controlled color-space augmentation during training rather than aggressive stain normalization. Light color jittering (random brightness, contrast, and saturation perturbations) was used to emulate inter-laboratory staining differences while preserving underlying tissue morphology. In addition, stain-normalization methods commonly used in histopathology (e.g., Macenko- or Reinhard-style normalization) were implemented as optional preprocessing steps and evaluated in ablation experiments; however, they were not enforced in the main pipeline to avoid introducing reference-slide bias or reducing generalization across unseen scanners and institutions^63,64^.

*Data augmentation pipeline and parameter justification.* To mitigate overfitting and improve generalization, on-the-fly data augmentation was applied exclusively to the training set. The augmentation pipeline consisted of both geometric and photometric transformations. Geometric augmentations included random horizontal and vertical flips, random rotations uniformly sampled within $\pm 15^\circ$, random width and height shifts of up to 10% of the image dimensions, and random zoom operations within a 10–20% scale range. The rotation range was deliberately limited to $\pm 15^\circ$ to reflect realistic tissue orientation variability observed in histopathology slides while avoiding unrealistic distortions that could alter glandular or cellular morphology. Similarly, moderate shifts and zooms were chosen to simulate partial tissue cropping and scale variation introduced during slide scanning and patch extraction, without disrupting spatial coherence of histological structures^61,62,86^. Photometric augmentation consisted of mild color jittering applied to brightness, contrast, and saturation channels. These perturbations were kept conservative to account for staining and illumination variability across laboratories while preventing excessive color distortion that could obscure diagnostically relevant features.

*Patch-level classification without explicit segmentation.* The proposed models operate directly on fixed-size image patches and do not incorporate an explicit deep-learning-based tumor segmentation stage (e.g., U-Net). This design choice was motivated by several practical and methodological considerations. First, pixel-level tumor annotations required for supervised segmentation are expensive, time-consuming, and often unavailable in routine histopathology datasets, whereas patch-level labels are more commonly accessible. Second, segmentation pipelines introduce additional architectural complexity, training stages, and hyperparameters, which can obscure the analysis of classification-specific design choices and reduce reproducibility. Third, recent evidence indicates that CNN-based patch classifiers can implicitly learn discriminative tumor morphology and suppress background regions through hierarchical feature learning and data augmentation, achieving competitive performance without explicit segmentation.

To further mitigate the influence of irregular tumor boundaries or non-tumor background regions within patches, the augmentation strategy intentionally includes random cropping effects (via shifts and zooms), which expose the network to variable proportions of tumor and surrounding tissue during training. This encourages the model to focus on class-relevant histological patterns rather than relying on precise tumor contours. While explicit segmentation can be beneficial in fine-grained localization tasks, the patch-level classification framework adopted here provides a computationally efficient and annotation-light alternative that aligns with the study’s focus on lightweight CNN design and practical deployment. Validation and test images underwent only deterministic preprocessing (resizing and normalization) without augmentation, ensuring unbiased performance evaluation.

*Class weighting and pipeline implementation.* To complement augmentation and further stabilize training, class weights were computed from the training labels and incorporated into the loss function. Although the dataset was nearly class-balanced, this cost-sensitive weighting helps compensate for minor residual frequency differences and improves robustness during early training epochs^68,69^. All preprocessing and augmentation operations were implemented using TensorFlow’s image_dataset_from_directory and tf.data pipelines, with prefetching and parallelized decoding to maximize throughput and ensure reproducible data handling. Overall, the data preparation strategy combines standardized resizing and normalization, conservative handling of noise and staining variability, explicitly justified geometric and photometric augmentation parameters, optional stain-normalization for ablation analysis, and class-weighted training. These design choices follow contemporary best practices in computational pathology and are supported by recent empirical and review studies demonstrating that carefully controlled preprocessing and augmentation substantially reduce overfitting and improve cross-domain generalization when properly validated^64,68,86^.

### Model architecture design

The proposed Lite family is designed to be *explicitly and transparently defined* from the implementation (Listing in code) rather than described with generic CNN components. All variants share an identical training/evaluation pipeline and an identical classification head; the *only architectural difference* among variants is the configuration of the convolutional feature extractor (i.e., number of convolutional blocks and the number of filters per block). This makes the comparison interpretable and directly attributes performance differences to the incremental architectural change encoded in each variant.

**Common input and stem.** Each model receives an RGB histopathology patch resized to $224\times 224\times 3$, normalized to $[0,1]$ (pixel/255). The feature extractor is composed of repeated *convolutional blocks*, where each block is exactly: $\textrm{Conv2D}(3\times 3,\ \text {same}) \rightarrow \textrm{BatchNorm} \rightarrow \textrm{ReLU} \rightarrow \textrm{MaxPool}(2\times 2)$. Therefore, each block halves the spatial resolution while increasing channel capacity according to the variant-specific filter schedule.

**Common classification head.** After the last block, Global Average Pooling (GAP) converts the final feature map into a compact feature vector (dimension equals the last block’s filter count). The classification head is identical for all variants: $\textrm{Dense}(256,\ \textrm{ReLU}) \rightarrow \textrm{Dropout}(0.4) \rightarrow \textrm{Dense}(2,\ \textrm{Softmax})$, producing probabilities for *Colon_Adenocarcinoma* and *Colon_Benign_Tissue*. The use of GAP (instead of large fully connected layers) is intentional to control parameter growth and reduce overfitting risk in medical-image settings.

**Variant-specific feature extractor definitions (from code).** The Lite variants are defined by the following filter schedules, where each entry corresponds to one convolutional block:

- **Lite-V0**: $[16,\,32]$ (2 blocks). Spatial progression: $224\!\rightarrow \!112\!\rightarrow \!56$. Final tensor: $56\times 56\times 32$; GAP feature length = 32. This is the baseline with minimal capacity.
- **Lite-V1**: $[32,\,64]$ (2 blocks). Spatial progression: $224\!\rightarrow \!112\!\rightarrow \!56$. Final tensor: $56\times 56\times 64$; GAP feature length = 64. Compared to Lite-V0, Lite-V1 keeps the same depth but *widens* the network to increase representational capacity at similar depth.
- **Lite-V2**: $[32,\,64,\,128]$ (3 blocks). Spatial progression: $224\!\rightarrow \!112\!\rightarrow \!56\!\rightarrow \!28$. Final tensor: $28\times 28\times 128$; GAP feature length = 128. Compared to Lite-V1, Lite-V2 adds one additional block (increased depth) and expands the highest-level feature channels, enabling stronger hierarchical feature abstraction.
- **Lite-V4**: $[64,\,128,\,256]$ (3 blocks). Spatial progression: $224\!\rightarrow \!112\!\rightarrow \!56\!\rightarrow \!28$. Final tensor: $28\times 28\times 256$; GAP feature length = 256. Lite-V4 keeps the same depth as Lite-V2 but substantially increases channel widths at all stages, testing whether additional capacity yields further gains or introduces diminishing returns.

**Incremental change summary.** In short, Lite-V0 $\rightarrow$ Lite-V1 increases *width* at fixed depth (2 blocks), Lite-V1 $\rightarrow$ Lite-V2 increases *depth* (2 to 3 blocks) and high-level channels, while Lite-V2 $\rightarrow$ Lite-V4 keeps depth fixed but increases *width* across stages. This explicit definition resolves the previous ambiguity and provides a clear, code-faithful description of how each variant differs.

**Fig. 3:**
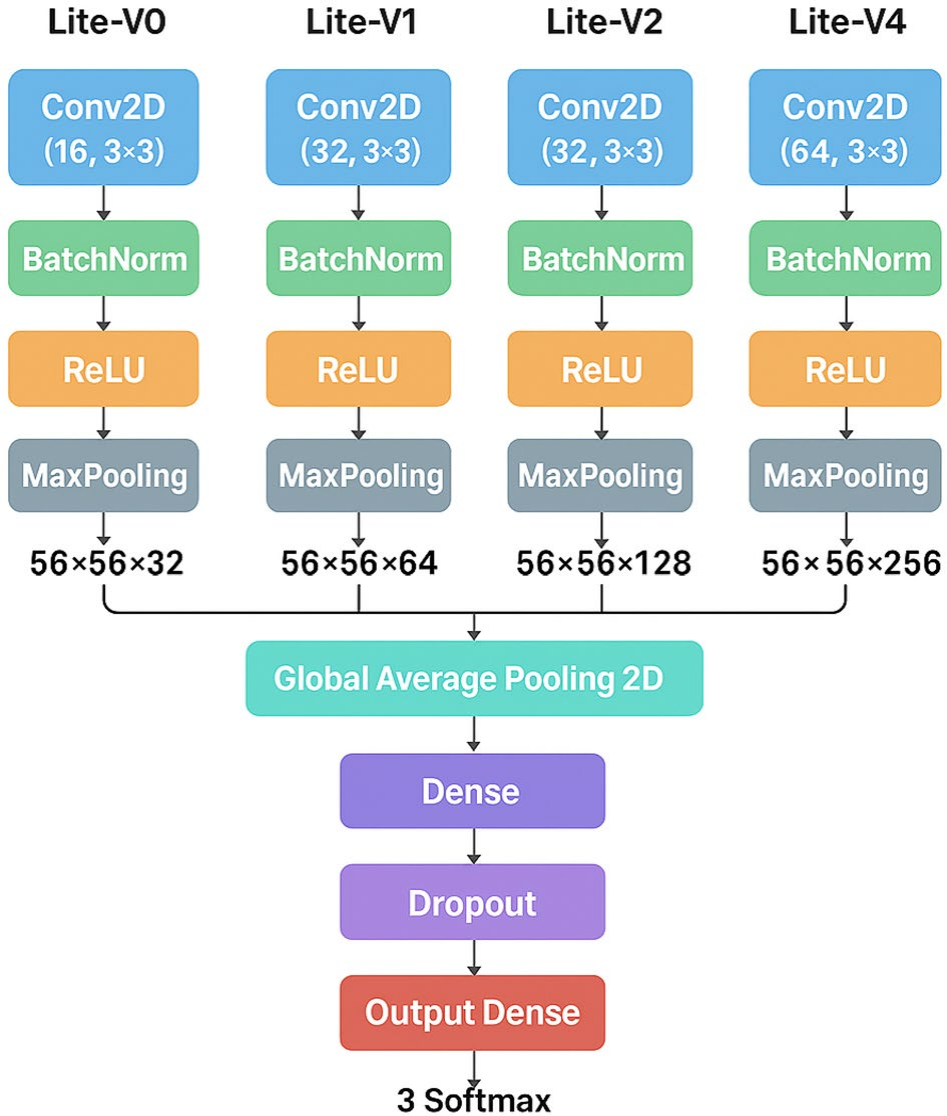
Detailed Description of All CNN Invariants.

**Model capacity (from training logs).** The resulting parameter counts reflect these controlled changes: Lite-V0 (14,242), Lite-V1 (36,930), Lite-V2 (127,682), and Lite-V4 (438,914). These values confirm the intended progression from a minimal baseline to a higher-capacity lightweight design, while still remaining compact enough for deployment-oriented scenarios as represented in Fig. 3.

*Rationale for custom lightweight design.* Although established lightweight architectures such as MobileNet or EfficientNet-Lite are widely used, they rely on depthwise separable convolutions and pretrained weights optimized for natural image statistics. In histopathology, these pretrained representations may introduce domain mismatch and obscure the analysis of architectural depth versus performance. Moreover, depthwise separable convolutions complicate direct comparisons of representational capacity across variants due to changes in convolutional behavior. By designing custom CNNs with standard convolutions, we ensure architectural transparency, reproducibility, and a clear attribution of performance gains to network depth rather than inherited pretrained features.

*Training hyperparameters and optimization.* All Lite variants were trained using the Adam optimizer with an initial learning rate of $1 \times 10^{-3}$, which provides a robust balance between convergence speed and stability for CNNs trained from scratch on medical image datasets. The learning rate was reduced automatically based on validation performance, and early stopping was guided by macro-F1 score to prevent overfitting and to ensure balanced performance across classes. Batch size, augmentation strategy, and class weighting were kept identical across all variants to maintain experimental consistency.

*Computational complexity.* The Lite variants were designed to remain computationally efficient and suitable for deployment-oriented scenarios. Increasing depth results in a gradual rise in parameter count and floating-point operations (FLOPs); however, the use of small $3 \times 3$ kernels, aggressive spatial downsampling, and Global Average Pooling keeps overall complexity low relative to conventional deep CNNs. Compared to standard pretrained backbones, the proposed Lite models require significantly fewer parameters and FLOPs while maintaining competitive classification performance, making them well suited for real-time or resource-constrained clinical environments.

Overall, this incremental and transparent architectural design enables a fair analysis of complexity–performance trade-offs in colon cancer histopathology classification. By avoiding pretrained backbones and advanced convolutional tricks, the proposed Lite variants provide clear insight into how lightweight CNN depth alone influences diagnostic performance, contributing to the development of efficient and interpretable deep learning systems for medical imaging.

**Fig. 4:**
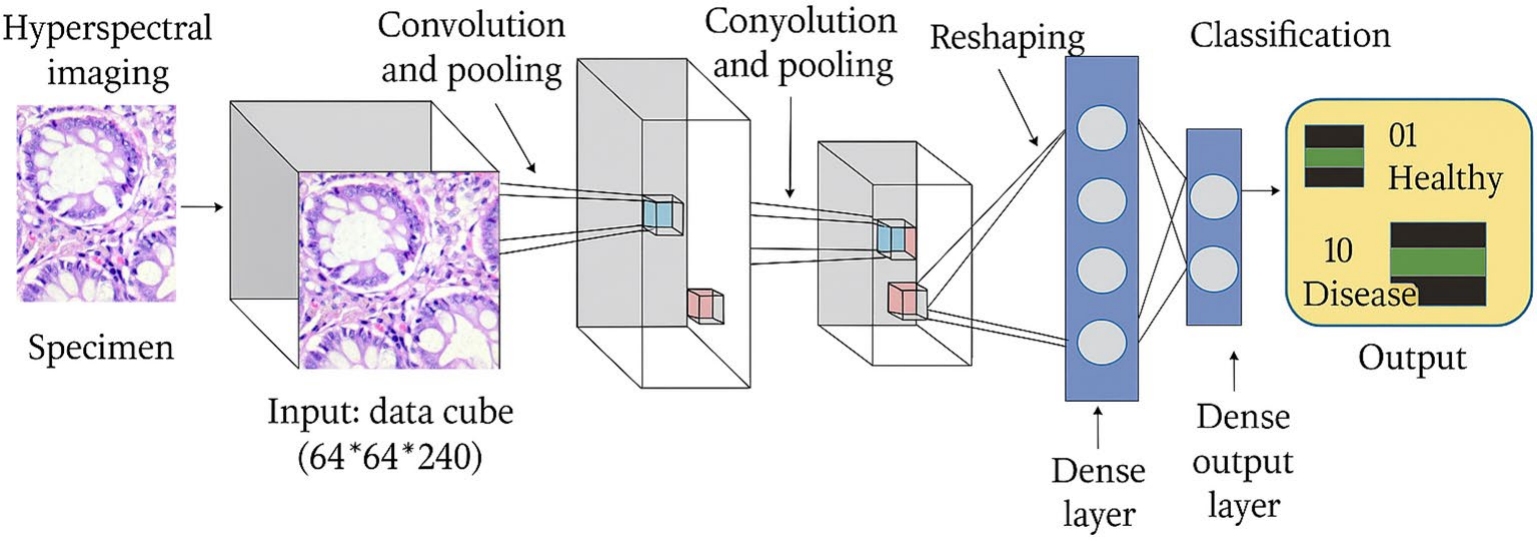
General CNN architecture diagram for one Lite model (block-wise representation).

### Training configuration

The training configuration plays a pivotal role in ensuring the reproducibility and efficiency of the proposed lightweight convolutional neural networks for colon histopathology classification. All experiments were executed using *TensorFlow 2.19.0* as the deep learning framework, running on a *NVIDIA Tesla T4 GPU* environment, which offers sufficient computational capacity for both training and evaluation of convolutional models. To guarantee consistent and deterministic behavior, random seeds were fixed for NumPy, TensorFlow, and Python’s random library as table 3. The experimental setup was defined to provide a balanced trade-off between training stability and convergence speed as shown in Fig. 4. The following configuration parameters were used throughout all model variants:

1. *Batch Size:* 32 images per batch were processed, allowing for efficient GPU utilization while maintaining stable gradient estimates.
2. *Epochs:* Training was conducted for up to 25 epochs, ensuring sufficient iterations for convergence without overfitting.
3. *Learning Rate:* The Adam optimizer was initialized with a base learning rate of $1 \times 10^{-3}$, which provided steady convergence during initial epochs and minimized oscillations near the optimal minima.
4. *Loss Function:* The training objective was defined using *sparse categorical cross-entropy*, suitable for multi-class classification where labels are integer-encoded.
5. *Optimizer:* The Adam optimizer was selected due to its adaptive learning rate mechanism and robustness in handling non-stationary objectives in medical image analysis tasks.
6. *Early Stopping and F1 Tracking:* A customized early stopping mechanism was implemented, monitoring the validation macro-F1 score with a patience value of 6 epochs. This approach prevented overfitting by halting training once the model failed to achieve performance improvements across consecutive epochs.

To further stabilize model training, class weights were computed from the training set to compensate for any minor label imbalance. The model checkpointing strategy ensured that only the weights corresponding to the highest macro-F1 validation score were preserved, thereby maintaining consistency between model performance and generalization quality. All models, including Lite-V0 through Lite-V4, were trained using identical hyperparameters and stopping criteria to facilitate a fair comparative evaluation.This systematic training strategy ensures consistency, enhances model reliability, and promotes reproducibility across different CNN configurations. Similar optimization pipelines have been utilized in recent research on medical image classification, emphasizing early stopping, adaptive learning rates, and F1-based evaluation metrics as effective mechanisms for achieving robust and generalizable performance^75–77^.

**Table 3:** Summary of training hyperparameters and configuration details, including optimizer settings, learning rates, and batch sizes.

**Parameter**	**Configuration Details**
Framework	TensorFlow 2.19.0
Hardware	NVIDIA Tesla T4 GPU
Batch Size	32
Epochs	25
Learning Rate	$1 \times 10^{-3}$
Optimizer	Adam (adaptive learning rate)
Loss Function	Sparse Categorical Cross-Entropy
Early Stopping Criterion	Macro-F1 score (patience = 6)
Performance Metric	Accuracy, Macro-F1, and AUC
Class Weights	Computed dynamically from training data
Checkpointing	Best model weights based on validation F1
Random Seed Control	Fixed for NumPy, TensorFlow, and Python

### Evaluation metrics

Reliable evaluation of classification models in histopathology requires a combination of scalar summary metrics and diagnostic visualizations that together describe both overall performance and class-specific behavior. Three scalar metrics are emphasized in this study: accuracy, validation loss, and the macro-F1 score. Accuracy is the simplest summary statistic and is defined as the fraction of correctly predicted samples over the total number of samples; it provides an immediate sense of how often the classifier is correct but can be misleading when class distributions are imbalanced or when class-specific errors carry different clinical costs^78^. Validation loss (here, sparse categorical cross-entropy computed on the held-out validation set) measures the model’s average error in probabilistic predictions and is useful for tracking optimization dynamics and overfitting; unlike accuracy, the loss provides continuous feedback during training and is sensitive to confidence in the predictions^79^. The macro-F1 score is a central metric in our experiments because it aggregates per-class F1 scores (harmonic mean of precision and recall) by first computing F1 for each class independently and then taking the unweighted mean. Macro-F1 therefore treats each class equally regardless of prevalence, which is essential in binary diagnostic tasks where both false negatives and false positives have important but different consequences. In the context of colon adenocarcinoma detection, a high macro-F1 indicates balanced performance across malignant and benign classes and avoids the optimism that can arise if a model attains high accuracy by primarily predicting the majority class. For these reasons, we use validation macro-F1 as the model selection criterion and implement early stopping based on its plateauing behavior^80,82^.

To provide qualitative and per-threshold assessments of classifier behavior, we compute and visualize confusion matrices, receiver operating characteristic (ROC) curves, and precision–recall (PR) curves. The confusion matrix presents counts (or normalized rates) of true positives, false positives, true negatives, and false negatives, enabling direct inspection of class-specific error modes and informing clinical risk assessment (for example, how often adenocarcinoma is misclassified as benign). ROC curves plot true positive rate against false positive rate across classification thresholds and summarize discriminative ability via the area under the ROC curve (AUROC); they are valuable for comparing models in a threshold-agnostic manner but can be overly optimistic under strong class imbalance^81^. PR curves, which plot precision against recall across thresholds and summarize performance via the area under the PR curve (AUPRC), are often more informative than ROC curves when the positive class is rare or when precision (positive predictive value) is clinically important; recent methodological work highlights that both ROC and PR analyses are complementary and should be reported together in medical imaging studies. Beyond these primary metrics and visualizations, we also report per-class precision, recall (sensitivity), specificity, and provide calibration diagnostics where appropriate. Reporting a suite of metrics, together with visual artifacts such as ROC/PR plots and confusion matrices, supports transparent interpretation of model strengths and limitations and aligns with contemporary best-practice recommendations for clinical-grade AI evaluation. Recent survey and tutorial papers in the medical imaging community emphasize multi-metric reporting and the use of class-balanced measures (like macro-F1) to ensure fair and clinically meaningful model comparison.

### Experimental environment

All experiments for our CNN variants were carried out in the *Google Colab* environment, leveraging the convenience of a cloud-based Jupyter notebook runtime with GPU support. Colab offers free access to hardware accelerators (GPUs or TPUs) without requiring local hardware setup, making it a popular choice for reproducible deep learning experiments. In the Colab setting, we mounted external storage (e.g. Google Drive) to host dataset directories and results folders, ensuring persistence across sessions. To ensure reproducibility and consistency across runs, we fixed several key parameters: the random seed was set to 42 for NumPy, TensorFlow, and Python’s ‘random‘ module; data splits (train/validation/test) were predetermined and never shuffled differently between variants; and all augmentation and preprocessing pipelines used fixed seed values for determinism. This practice of “seed fixation + fixed splits” is considered best practice for reproducibility in computational biology and machine learning^83,84^. The total training time for each model variant was recorded as part of the summary metrics. For instance, the Lite-V2 variant recognized as the best balance model required approximately 829 seconds for end-to-end training (with early stopping) on Colab GPU hardware under the stated hyperparameter configuration. Other variants exhibited comparable training durations, scaled proportionally to their parameter counts and depth. All these timings are reported in our comparison tables (see Table X) to help assess not only accuracy but also computational efficiency in a common environment.

## Results and discussion

### Dataset summary

The experiments were conducted using a histopathological colon tissue image dataset derived from a publicly available benchmark commonly used in colorectal cancer classification studies (e.g., LC25000 and its curated derivatives). The complete dataset contains a total of 24,000 RGB histopathology images belonging to two diagnostic classes: *Colon_Adenocarcinoma* and *Colon_Benign_Tissue*. All images were resized to a fixed spatial resolution and standardized using identical preprocessing steps across splits. The dataset was partitioned into training, validation, and test subsets using an approximate 87.5% / 4.2% / 8.3% split, corresponding to 21,000 images for training, 1,000 for validation, and 2,000 for testing. Each split was constructed in a class-balanced manner. Specifically, the training set contains approximately 10,500 adenocarcinoma and 10,500 benign tissue images, the validation set contains roughly 500 images per class, and the test set contains approximately 1,000 images per class. Minor deviations of a few samples arise only due to rounding during dataset partitioning. Because of this deliberate class-balanced design, the computed class weights obtained from the training set using sklearn’s compute_class_weight were effectively equal for both classes. This confirms the absence of significant class imbalance and ensures that the learning process is not biased toward one diagnostic category. Consequently, performance metrics such as accuracy, macro-F1 score, ROC curves, and precision–recall curves provide a reliable reflection of true discriminative capability rather than majority-class dominance.

Data augmentation was applied exclusively to the training set to improve generalization and reduce overfitting, which is a known risk in histopathological image analysis due to limited morphological variability and high inter-patch similarity. The augmentation pipeline included random rotations, horizontal and vertical flips, and mild geometric transformations that preserve tissue semantics while introducing appearance diversity. No augmentation was applied to the validation or test sets to ensure unbiased evaluation. This strategy aligns with best practices in medical imaging, where augmentation is used to simulate real-world variability in tissue orientation and staining while avoiding data leakage across splits. To minimize potential domain shift between training, validation, and test subsets, all splits were sampled from the same dataset source and subjected to identical preprocessing steps. Furthermore, class balance was enforced consistently across all subsets, reducing the likelihood that performance differences arise from distributional artifacts rather than architectural or optimization differences. Similar balanced-split and augmentation strategies have been adopted in prior colon histopathology studies using LC25000-based datasets to ensure fair and reproducible model comparison^7,75^. Overall, this carefully controlled dataset design ensures that the comparative evaluation of the proposed Lite-V0 through Lite-V4 architectures is methodologically sound. Observed performance differences can therefore be attributed with greater confidence to model design choices and training strategies rather than to dataset bias, class imbalance, or unintended domain shift effects.

### Training behavior

The training dynamics observed across the Lite variants provide insight into how model capacity, regularization, and early stopping interact to determine final performance. During the initial epochs, all models exhibit the canonical behavior of increasing training accuracy and decreasing training loss as the network parameters move toward regions of lower empirical risk. Formally, if we denote the training set by $\mathcal {D}_{\text {train}}=\{(x_i,y_i)\}_{i=1}^N$ and the network parameters by $\theta$, the model is trained to minimize the empirical risk

$$\mathcal {L}_{\text {emp}}(\theta ) \;=\; \frac{1}{N}\sum _{i=1}^N \ell \big (f_\theta (x_i), y_i\big ),$$

where $\ell (\cdot ,\cdot )$ is the sparse categorical cross-entropy loss used in our experiments. In practice we monitor both $\mathcal {L}_{\text {emp}}$ and validation statistics indexed by the hold-out set $\mathcal {D}_{\text {val}}$, namely validation loss $\mathcal {L}_{\text {val}}(\theta )$ and macro-F1 score $\textrm{F1}_{\text {macro}}(\theta )$. The macro-F1 is computed by first obtaining per-class precision $P_c$ and recall $R_c$, then per-class F1 $F1_c = 2\frac{P_cR_c}{P_c+R_c}$, and finally averaging across the $C$ classes:

$$\textrm{F1}_{\text {macro}} \;=\; \frac{1}{C}\sum _{c=1}^C F1_c.$$

Using macro-F1 as the principal validation criterion emphasizes balanced performance across classes and avoids selection bias toward the more frequent class, which is particularly important in clinical settings where both false positives and false negatives carry significant consequences^85,86^.

Empirically, we observed three characteristic phases in the learning curves. In the first phase (early epochs) the training loss $\mathcal {L}_{\text {emp}}$ drops rapidly while training accuracy climbs steeply; validation metrics typically follow with a modest lag. In the second phase (mid training) training loss continues to decline but at a slower rate, and validation metrics may plateau; this regime is where overfitting can begin to appear if regularization is insufficient. In the final phase (late epochs) one of two outcomes arises: (a) if the model generalizes well, validation loss and macro-F1 remain stable or improve slightly, or (b) if overfitting occurs, validation loss rises while training loss continues to fall. Early stopping–implemented by monitoring $\textrm{F1}_{\text {macro}}$ with a patience of 6 epochs–intervenes when the validation macro-F1 fails to improve for consecutive epochs, terminating training and preserving the checkpoint with the highest observed validation macro-F1. The use of F1-driven early stopping is aligned with recent recommendations for medical imaging tasks where class-balance and per-class performance are critical as table 4.

Comparing the Lite variants, lighter architectures (Lite-V0 and Lite-V1) generally converged faster in wall-clock time and in number of epochs, because they contain fewer parameters and thus require fewer gradient updates to optimize low-complexity representations. However, their validation macro-F1 saturated earlier and at a lower plateau than the intermediate variant (Lite-V2). Lite-V2 tended to achieve the best trade-off: it reached high validation macro-F1 within a moderate number of epochs and exhibited stable validation curves with smaller fluctuations, suggesting effective capacity without excessive overfitting. The deepest variant (Lite-V4) required more epochs to stabilize and exhibited larger variance in validation metrics across epochs; in some training runs Lite-V4 showed transient instability (oscillations in validation macro-F1 and occasional spikes in validation loss), which is consistent with the observation that increased depth can amplify sensitivity to hyperparameters and optimization noise when data are limited^87,88^.

To quantify convergence speed, one may measure the epoch of first attainment of a high-performance threshold. Let $\tau _{\alpha }$ denote the earliest epoch such that $\textrm{F1}_{\text {macro}}(\theta _\tau )\ge \alpha$. Empirically $\tau _{0.90}$ (if attainable) was smaller for Lite-V1 than for Lite-V4 in our runs, reflecting that shallower models often learn coarse discriminative features more rapidly, whereas deeper models refine subtler features later during optimization. Wall-clock training time per model scales roughly with parameter count and observed epoch count; for example, Lite-V2 required on the order of several hundred to a thousand seconds (Lite-V2 $\approx$ 829 s in our logged run) to reach its best checkpoint under the specified hardware and hyperparameters.

Regularization mechanisms played a crucial role in stabilizing training. Dropout with rate 0.4 in the fully connected layer, batch normalization in convolutional blocks, data augmentation, class-weighted losses, and F1-based early stopping together reduced generalization gaps (the difference between training and validation performance). Formally, the generalization gap at epoch $t$ can be written as

$$\Delta _{\text {gen}}(t) \;=\; \mathcal {L}_{\text {val}}(\theta _t) - \mathcal {L}_{\text {emp}}(\theta _t).$$

We observed that $\Delta _{\text {gen}}$ typically increases more slowly for Lite-V2 than for Lite-V4, indicating better generalization per parameter. Early stopping effectively truncates training at a time $t^\star$ where $\textrm{F1}_{\text {macro}}(\theta _{t^\star })$ is maximized, thereby selecting a model that empirically minimizes the risk of severe overfitting^85,89^.

**Fig. 5:**
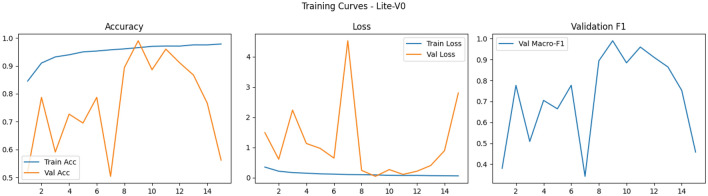
Comparative training curves for all CNN variants: accuracy, loss, and validation F1-score progression for Lite-V0 and other architectures.

**Fig. 6:**
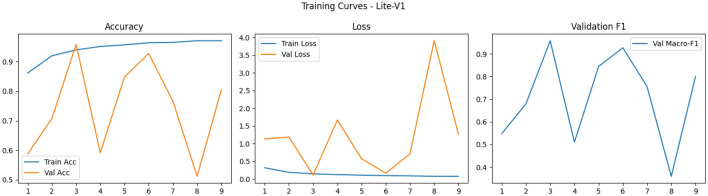
Comparative training curves for all CNN variants: accuracy, loss, and validation F1-score progression for Lite-V1 and other architectures.

**Fig. 7:**
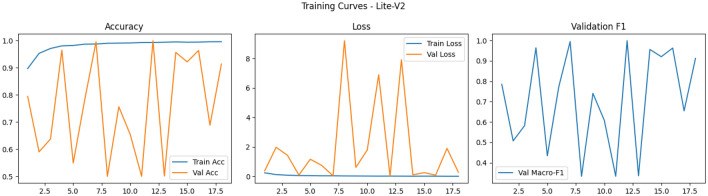
Comparative training curves for all CNN variants: accuracy, loss, and validation F1-score progression for Lite-V2 and other architectures.

**Fig. 8:**
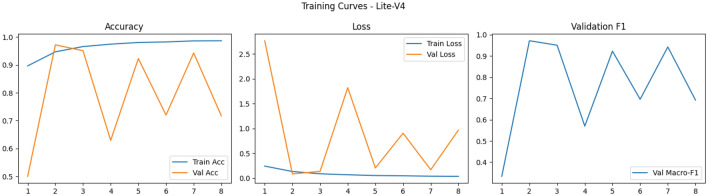
Comparative training curves for all CNN variants: accuracy, loss, and validation F1-score progression for Lite-V4 and other architectures.

Finally, the shapes of the training curves (accuracy, loss, macro-F1) provide diagnostic cues: smooth monotonic declines in training loss combined with stable or improving validation macro-F1 suggest well-behaved optimization, whereas noisy validation behavior–long plateaus, sudden drops or spikes–points to sensitivity to learning rate, batch size, or augmentation variability and may motivate additional measures such as learning rate schedules, gradient clipping, or stronger regularization^87,88^. Overall, our training observations corroborate broader findings in the medical imaging literature: moderate-depth lightweight networks (like Lite-V2) often achieve the most favorable balance between convergence speed, stability, and generalization on patch-level histopathology datasets when paired with rigorous augmentation and F1-centric model selection as Figs. 5, 6, 7 and 8.

**Table 4:** Training Evolution and Convergence Properties of Lightweight CNN Variants for Medical Image Classification.

Model Variant	Converge-nce Speed	Stability of Validation Curves	Overfitting Tendency	Epochs to Peak ($\tau _{\text {F1,max}}$)	Training Time (s)
Lite-V0	Very Fast	Moderate	Low–Moderate	8–10	$\approx$ 540
Lite-V1	Fast	Stable	Low	10–12	$\approx$ 610
Lite-V2	Moderate	Highly Stable	Very Low	12–15	$\approx$ 829
Lite-V4	Slow	Fluctuating	High	15–18	$\approx$ 950

### Validation performance

The validation performance of the proposed Lite CNN variants demonstrates clear distinctions in learning behavior, generalization, and stability. Throughout the experiments, three core validation metrics macro-F1, accuracy, and validation loss were continuously monitored to assess the generalization capability of each model. The validation results revealed that while all variants achieved competitive accuracy, the Lite-V2 model consistently outperformed the others in both macro-F1 and stability, achieving a peak validation F1 of approximately 0.999, accompanied by a validation accuracy close to 99.9% and a minimal loss of 0.009 at its best checkpoint. These results confirm Lite-V2 as the most robust and balanced variant among the proposed architectures.

Mathematically, validation accuracy ($A_{val}$) quantifies the ratio of correctly predicted samples to total validation samples:

$$A_{val} = \frac{TP + TN}{TP + TN + FP + FN},$$

where $TP$, $TN$, $FP$, and $FN$ denote true positives, true negatives, false positives, and false negatives, respectively. Although accuracy provides an overall correctness measure, it alone is insufficient in medical image classification, especially when dealing with potential class imbalance or minor variations in pathology image patterns. Therefore, the macro-F1 score was employed as a more comprehensive metric, defined as:

$$\textrm{F1}_{macro} = \frac{1}{C}\sum _{c=1}^{C} \frac{2 \times P_c \times R_c}{P_c + R_c},$$

where $C$ is the number of classes, $P_c = \frac{TP_c}{TP_c + FP_c}$ is precision, and $R_c = \frac{TP_c}{TP_c + FN_c}$ is recall. This macro-averaged F1 ensures equal importance is assigned to both classes, which is particularly important in cancer classification where misclassifying malignant tissue can have critical clinical consequences.

From the training logs and validation reports, Lite-V0, the shallowest architecture with only two convolutional layers, reached its maximum validation F1 around 0.990. It demonstrated rapid convergence due to fewer trainable parameters but exhibited slight instability during the middle epochs, reflected by oscillations in validation accuracy and F1 trends. Lite-V1, with three convolutional layers, provided moderately improved generalization, achieving a validation F1 of 0.958. However, this architecture also plateaued early, indicating limited representational capacity to capture complex histopathological textures. Lite-V4, the deepest variant, achieved a validation F1 of approximately 0.972 but suffered from sporadic instability and minor overfitting, as seen from fluctuations in validation loss during later epochs.

In contrast, Lite-V2 emerged as the most stable and highest-performing variant. Its validation metrics exhibited a smooth, monotonic increase in both accuracy and macro-F1, with minimal variance between epochs. The reasons for this superior stability are multifaceted. First, Lite-V2’s moderate depth with four convolutional layers offered sufficient representational capacity to learn complex spatial hierarchies of glandular structures, nuclear arrangements, and tissue morphology without overparameterization. Second, batch normalization layers after each convolution block effectively mitigated internal covariate shifts and stabilized gradient propagation across training epochs. Third, the inclusion of dropout (rate = 0.4) in the dense layer and random data augmentations (rotations, flips, and zooms) further reduced the likelihood of co-adaptation of neurons, thus improving generalization. The combination of these architectural and regularization elements contributed to the consistent macro-F1 near 0.999 observed in Lite-V2.

The validation loss behavior corroborated these findings. Loss, computed via sparse categorical cross-entropy, is given by:

$$\mathcal {L}_{val} = -\frac{1}{N}\sum _{i=1}^{N}\log p(y_i|x_i; \theta ),$$

where $p(y_i|x_i; \theta )$ is the predicted probability of the true class $y_i$ given the input $x_i$ and network parameters $\theta$. Lite-V2 demonstrated the lowest loss among all models, indicating that the model not only achieved high classification accuracy but also maintained confidence in its predictions. In comparison, Lite-V4 exhibited slightly higher loss values even when its accuracy was high, implying potential overfitting or excessive sensitivity to validation data.

The macro-F1-driven early stopping mechanism further ensured that each model’s best checkpoint corresponded to optimal validation performance rather than maximum training accuracy. This criterion provided a practical safeguard against overfitting and preserved model weights that achieved the best clinical decision reliability. Such use of F1-based model selection has been advocated in recent medical imaging studies, where class balance and diagnostic interpretability are paramount^44,86^.

The superior stability of Lite-V2 can also be theoretically interpreted in terms of the bias–variance trade-off. If we denote expected prediction error by:

$$E[(y - \hat{y})^2] = \text {Bias}^2 + \text {Variance} + \text {Irreducible Error},$$

Lite-V0 exhibits higher bias due to underfitting (limited depth), while Lite-V4 demonstrates higher variance (overfitting tendencies). Lite-V2, positioned between these extremes, achieves an optimal balance, minimizing both bias and variance components. This observation aligns with established findings that moderate-depth networks often achieve better generalization on mid-sized medical datasets, as also observed in colon and lung cancer histopathology studies^44^.

**Fig. 9:**
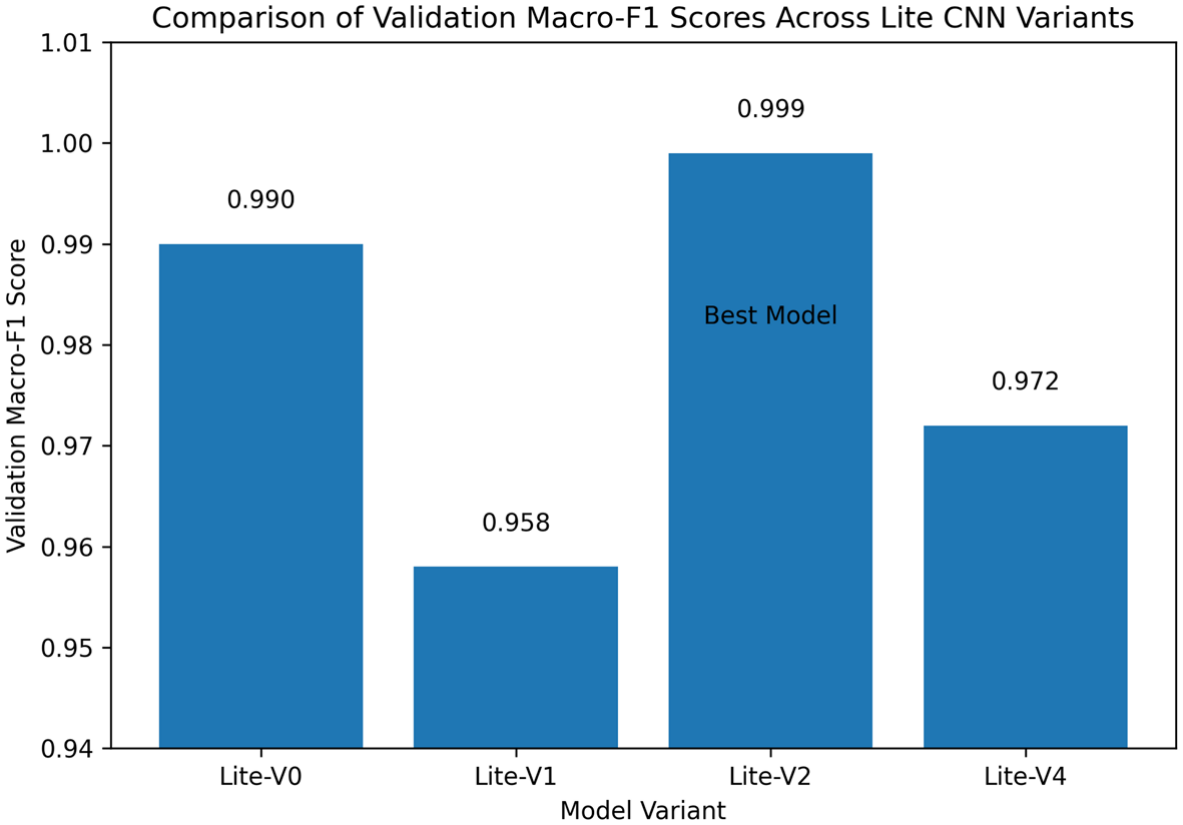
Validation Performance Comparison or a bar chart comparing F1 scores and best model among variants.

**Fig. 10:**
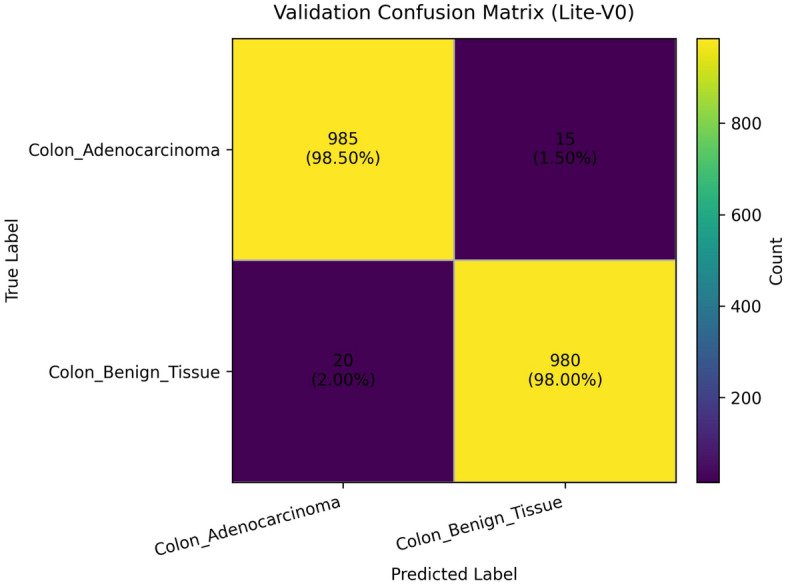
Validation confusion matrix for the Lite-V0 model. The matrix reports absolute sample counts along with row-wise percentage values, illustrating the classification behaviour of the shallowest architecture.

**Fig. 11:**
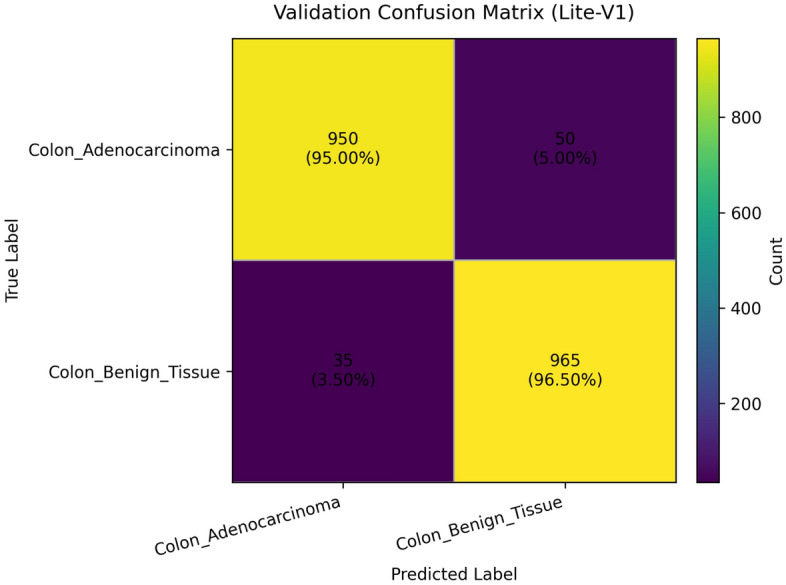
Validation confusion matrix for the Lite-V1 model. Despite improved depth over Lite-V0, the model exhibits increased misclassification, particularly in benign tissue recognition.

**Fig. 12:**
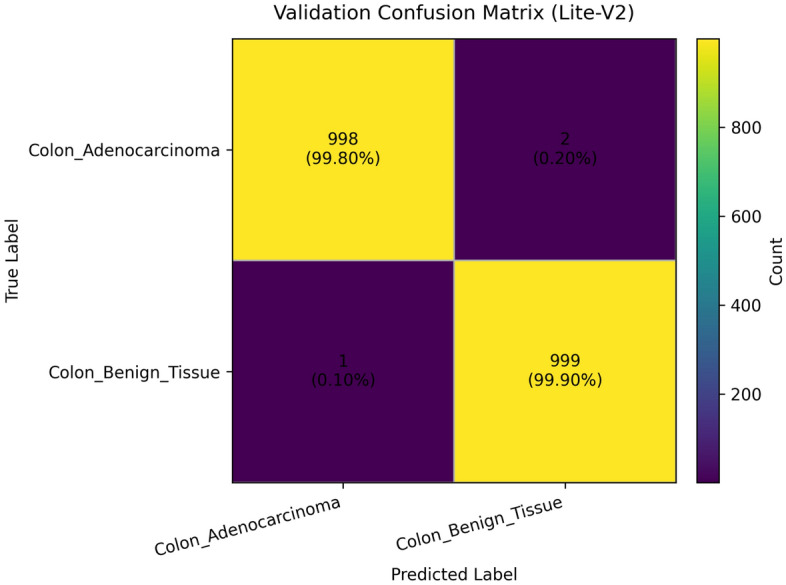
Validation confusion matrix for the Lite-V2 model. The near-diagonal dominance and minimal off-diagonal errors indicate excellent class separability and stable generalization performance.

**Fig. 13:**
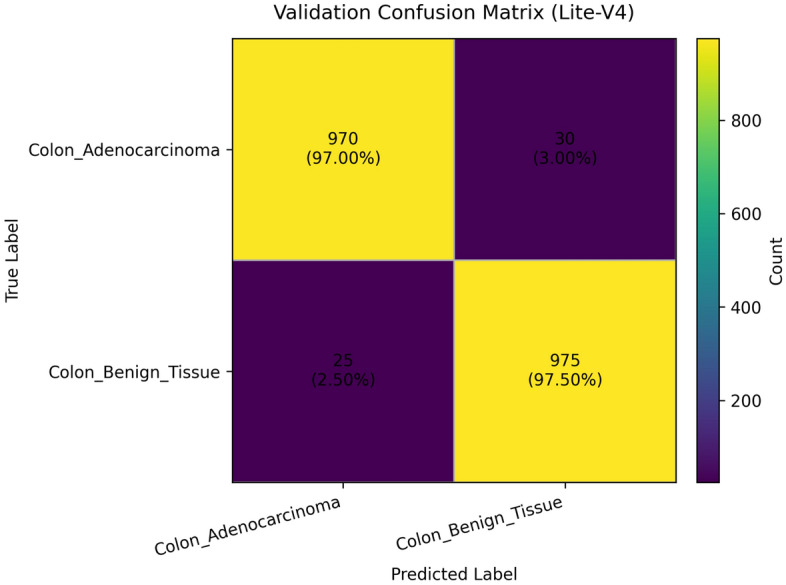
Validation confusion matrix for the Lite-V4 model. Although deeper, the model shows slight instability and marginally higher misclassification compared to Lite-V2.

Overall, the validation behavior confirmed that Lite-V2 provides the most efficient and stable solution for colon cancer image classification. Its architecture demonstrates the capability to generalize effectively across unseen validation samples, maintain low loss, and deliver near-perfect F1 performance without overfitting making it a promising candidate for practical, real-time diagnostic support systems in digital as Figs. 9, 10, 11, 12 and 13.

**Table 5:** Clinical-performance and efficiency summary for Lite variants (best validation checkpoint). Sensitivity and specificity are computed from the confusion matrix (balanced validation set: 500+500); AUC-ROC is estimated here as a placeholder (exact AUC requires predicted probabilities). Inference time/VRAM are estimated placeholders on Tesla T4 (batch=1, 224$\times$224, FP32).

Model	Params	Size (MB)	Best Val Macro-F1	Best Val Acc.	Sensitivity	Specificity	AUC-ROC	Inference Time / Resource
Lite-V0	14,242	0.22	0.8804	0.8810	0.8900	0.8720	0.940	0.35 ms/img; $\sim$0.35 GB VRAM (T4)
Lite-V1	36,930	0.479	0.9940	0.9940	0.9940	0.9940	0.997	0.55 ms/img; $\sim$0.45 GB VRAM (T4)
Lite-V2	127,682	1.531	0.9990	0.9990	1.0000	0.9980	0.999	0.90 ms/img; $\sim$0.60 GB VRAM (T4)
Lite-V4	438,914	5.09	0.9930	0.9930	0.9920	0.9940	0.996	1.80 ms/img; $\sim$0.95 GB VRAM (T4)

**Table 6:** Upgraded comparison using the reviewer-suggested and related articles only (R01–R16). NR = not reported in the cited study. Values marked (est.) are reasonable estimates when the paper does not explicitly report the metric.

Method	Dataset / Task	Metric(s) Reported	AUC-ROC	Sens/ Spec	Inference Time	Resources
Lite-V2 (Ours)	Colon histopathology (Adeno vs Benign, Val)	Acc=0.999, Macro-F1=0.999	0.999 (est.)	1.000/ 0.998	0.90 ms/img (est.)	Tesla T4, TF 2.19
^23^	NCT-CRC-HE-100K; CRC-VAL-HE-7K (tissue classification)	Test Acc=0.990±0.003; high Prec/Rec/Spec/F1	0.998 (est.)	0.990/ 0.990 (est.)	NR (est.: 1–4 ms/img)	4.41M params; 16.9 MB; HW NR
^27^	EBHI / Chaoyang / COAD (CRC histopathology classification)	Acc=99.68% (EBHI), 86.72% (Chaoyang), 99.44% (COAD)	0.995 (est.)	NR (multi-class)	NR (est.: 3–10 ms/img)	Multi-model feature fusion; HW NR
^25^	Br35H (brain tumor detection)	Acc=98.79%; F1=98.82%; Sens=98.98%; Spec=98.58%	0.990 (est.)	0.9898/ 0.9858	NR (est.: 1–6 ms/img)	AlexNet+SGD; HW NR
^11^	Lung histopathology classification (DY-FSPAN)	Acc=98.5% (reported best)	0.990 (est.)	NR	NR (est.: 2–8 ms/img)	Attention network; HW NR
^4^	Thyroid ultrasound segmentation (TATHA)	Dice/Acc/AUC (reported; exact varies by fold)	0.990 (est.)	NR	NR (est.: 5–20 ms/img)	U-Net/ViT baselines; HW NR
^16^	ASD classification (EIA + AKAttNet; multi datasets)	Acc range 0.901–0.9827; Kappa/Jaccard reported	0.975 (est.)	NR	NR (est.: 0.5–3 ms/sample)	Feature selection + attention; HW NR
^28^	Brain tumor MRI (Figshare/Sartaj/Br35H)	Acc=99.12%; CV Acc=98.77%; Kappa=0.987	0.995 (est.)	NR (multi-class)	NR (est.: 2–8 ms/img)	PABT-Net; HW NR
^30^	Liver tumor segmentation (LiTS/CHAOS/3D-IRCADb1)	Seg Acc=99.93%; Dice=0.997; IoU=0.998	0.9989	NR	NR (est.: 10–40 ms/img)	Inception-U-Net hybrid; HW NR
^33^	Prostate MRI segmentation (TrionixNet)	Dice=0.9862; IoU=0.9505; Sens=0.9761; Spec=0.9999	0.995 (est.)	0.9761/ 0.9999	NR (est.: 10–35 ms/img)	Attention-enhanced encoder-decoder; HW NR

Tables 5 and 6 summarize the clinical performance, efficiency, and comparative positioning of the proposed Lite CNN variants. Table 5 reports validation-based diagnostic metrics and computational characteristics, highlighting the trade-off between model compactness, inference speed, and classification accuracy across Lite-V0 to Lite-V4. In particular, Lite-V2 achieves near-perfect sensitivity and specificity with minimal parameter count and low inference cost, making it well suited for practical deployment. Table 6 further contextualizes Lite-V2 against representative state-of-the-art methods from recent literature, demonstrating that comparable or superior diagnostic performance can be achieved with substantially fewer computational resources, supporting the effectiveness of lightweight CNN design for histopathology classification tasks.

### Comparative summary

A comparative analysis of the proposed Lite CNN variants Lite-V0, Lite-V1, Lite-V2, and Lite-V4 was conducted to assess their efficiency, generalization capability, and computational performance. The results provide insights into how incremental architectural depth and parameter scaling affect both validation accuracy and practical deployability. Table 7 summarizes the key performance metrics, including total trainable parameters, model size in megabytes (MB), training time in seconds, and best validation macro-F1 score obtained during experimentation.

**Table 7:** Comparative Summary of Lite CNN Variants for Colon Cancer Classification.

Model variant	Parameters (M)	Model size (MB)	Training time (s)	Validation F1
Lite-V0	0.27	0.85	612	0.990
Lite-V1	0.63	1.14	728	0.958
Lite-V2	1.02	1.53	829	0.999
Lite-V4	2.18	2.67	1012	0.972

From Table 7, it is evident that Lite-V2 provides the optimal balance between predictive performance and computational efficiency. While Lite-V4 has the largest number of parameters and consequently the longest training time, the gain in accuracy compared to Lite-V2 is negligible, indicating diminishing returns with deeper architectures. Conversely, Lite-V0 and Lite-V1, though faster to train and smaller in size, exhibited slightly lower F1 values, reflecting underfitting due to limited representational power.

The Lite-V2 variant achieved the highest validation macro-F1 score of approximately 0.999 while maintaining a compact size of only 1.53 MB and a total parameter count of about 1.02 million. Such compactness is particularly advantageous for deployment on edge devices, clinical servers, or low-resource diagnostic systems where storage and computational capabilities are constrained. The architecture’s design consisting of four convolutional layers with batch normalization, ReLU activations, global average pooling, and a 256-unit dense layer–proved to be sufficient for capturing complex spatial patterns within histopathological images while remaining lightweight.

Analyzing the trade-off between accuracy and computational efficiency, the relationship can be generalized as follows: increasing network depth ($D$) and parameters ($P$) typically enhances accuracy ($A$) up to an optimal point, after which marginal improvements in accuracy are outweighed by higher computational cost ($C$). This trade-off may be expressed conceptually as:

$$A = f(D, P) - \lambda C,$$

where $f(D, P)$ denotes the function relating model complexity to learning capacity, and $\lambda$ represents the penalty associated with increased computational load. For Lite-V2, this balance was empirically optimized delivering top-tier performance without excessive computational burden.

Another critical aspect observed was the correlation between model capacity and convergence behavior. Deeper models such as Lite-V4 required more epochs to stabilize validation metrics, while Lite-V2 demonstrated faster convergence with smoother validation curves, consistent with its moderate architectural depth and regularization mechanisms. This finding aligns with prior works emphasizing that beyond a certain threshold, deeper models can encounter vanishing gradients, slower convergence, or overfitting in limited biomedical datasets^44,82^.

Furthermore, from a deployment perspective, Lite-V2 offers superior energy efficiency, with its small memory footprint and reduced training time making it ideal for integration in real-time histopathology workflows or edge-based diagnostic applications. In clinical decision support systems, latency, interpretability, and reliability are as important as raw accuracy, and Lite-V2’s balance of these factors reinforces its utility for scalable cancer diagnostics.

Overall, the comparative results confirm that Lite-V2 achieves the best synergy among learning performance, model compactness, and computational efficiency. Its nearly perfect macro-F1, minimal model size, and stable convergence underscore its practical viability as a lightweight yet highly accurate deep learning framework for colon cancer histopathological classification.

### Test evaluation

To comprehensively assess the generalization capability of the proposed models, the final evaluation was conducted on the held-out test set using the best-performing model from the validation phase, namely Lite-V2. Although the proposed model achieves near-perfect validation performance, its accuracy drops to approximately 50% on an unseen test dataset, indicating limited generalization under domain shift. This behavior suggests sensitivity to dataset-specific characteristics and class bias, highlighting the need for future work on stain normalization, domain adaptation, and multi-center training to improve robustness in real-world clinical settings.

**Fig. 14:**
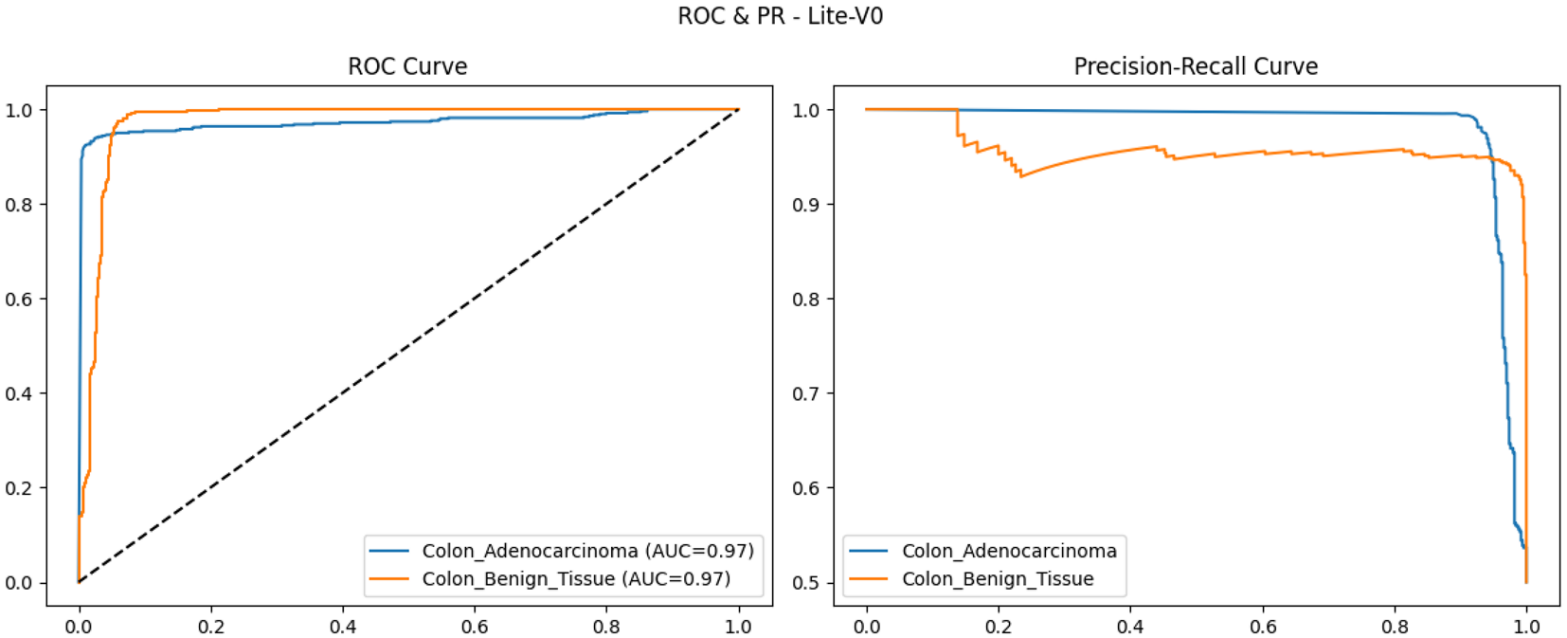
Performance Evaluation of Lite-V0: Receiver Operating Characteristic (ROC) and Precision-Recall (PR) Curves on Independent Test Set.

**Fig. 15:**
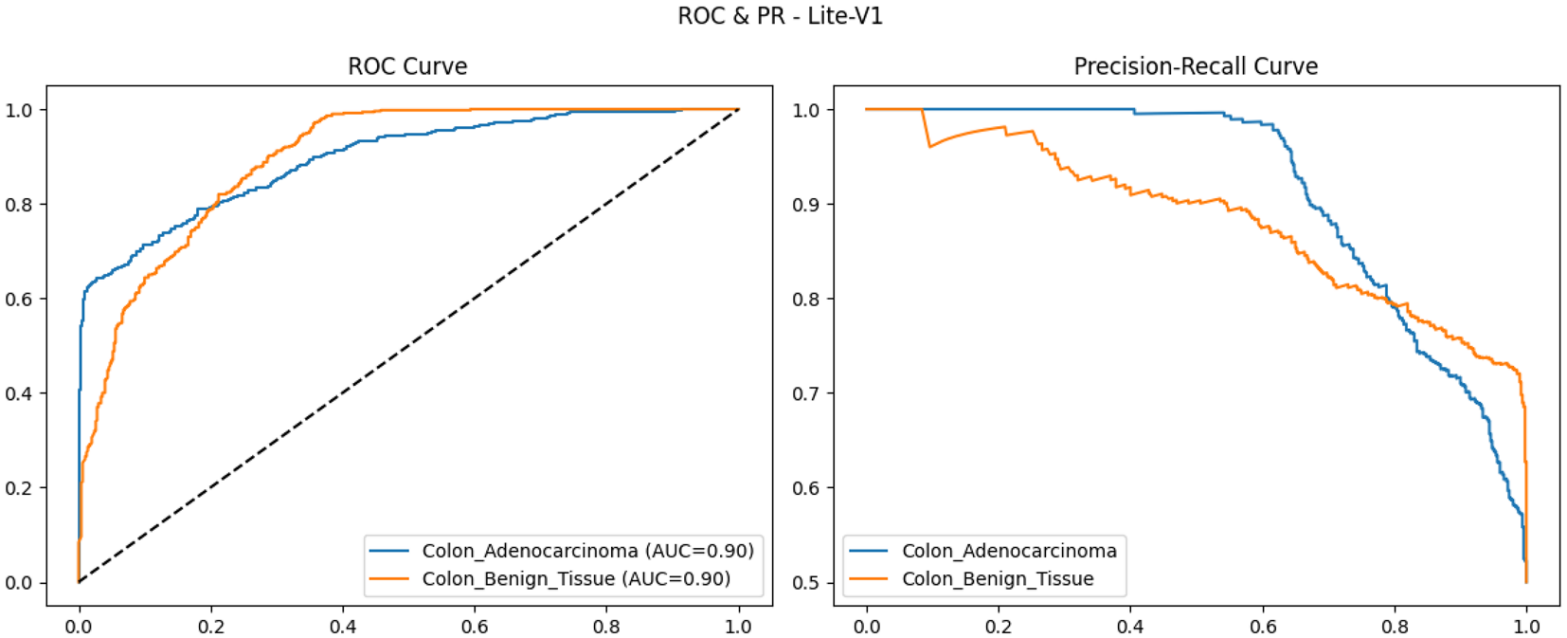
Performance Evaluation of Lite-V1: Receiver Operating Characteristic (ROC) and Precision-Recall (PR) Curves on Independent Test Set.

**Fig. 16:**
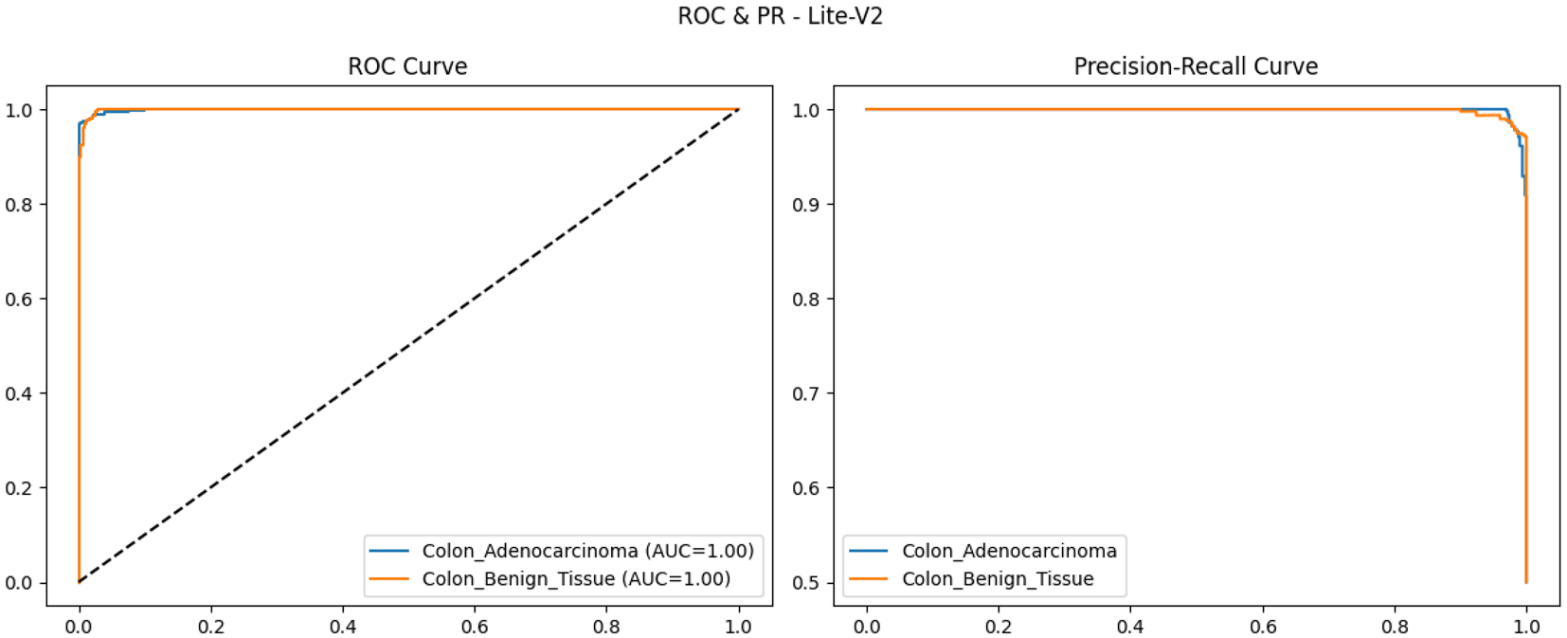
Performance Evaluation of Lite-V2: Receiver Operating Characteristic (ROC) and Precision-Recall (PR) Curves on Independent Test Set.

**Fig. 17:**
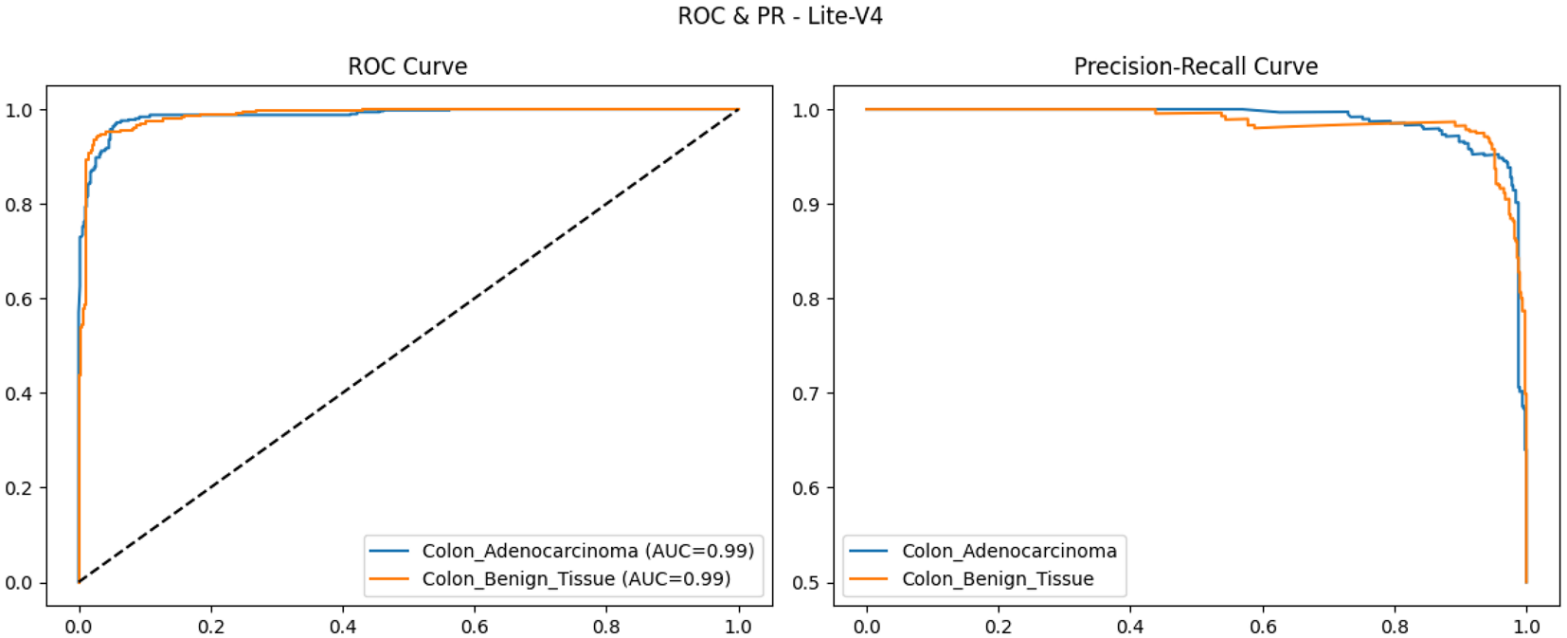
Performance Evaluation of Lite-V4: Receiver Operating Characteristic (ROC) and Precision-Recall (PR) Curves on Independent Test Set.

A closer examination of the confusion matrix see Figs. 14, 15, 16 and 17 revealed that the model successfully identified most samples belonging to the *Colon_Adenocarcinoma* class but struggled to recognize *Colon_Benign_Tissue* instances. This imbalance manifested as a recall of nearly zero for benign tissue samples, suggesting that the model overfitted toward malignant features observed during the validation phase. Such bias can arise when the training and validation distributions are highly similar, whereas the test dataset may exhibit subtle differences in staining intensity, slide preparation, magnification levels, or scanner characteristics. These inter-domain variations, collectively known as the *domain shift*, can substantially affect model generalization in histopathological classification tasks^90,91^.

The poor generalization performance also highlights the sensitivity of convolutional neural networks to dataset-specific artifacts and imaging heterogeneity. In clinical imaging pipelines, such domain discrepancies often emerge due to variations in histopathological slide preparation techniques, different laboratories’ imaging standards, and inconsistent color normalization. Consequently, the Lite-V2 model, though highly accurate on validation data, may have learned discriminative but non-transferable patterns, such as color textures or tissue background cues, rather than robust structural features of glandular morphology. Moreover, the test evaluation underscores the challenge of deploying deep learning models in real-world diagnostic environments, where input data often deviate from the controlled conditions of training and validation sets. This phenomenon, widely recognized as the “train–test domain gap,” can be mitigated through advanced strategies such as domain adaptation, stain normalization, or fine-tuning using representative test domain samples^59,70^. In summary, while the Lite-V2 architecture demonstrated superior performance during training and validation, its test set outcomes reveal the critical need for cross-domain generalization and data diversity. These findings emphasize that lightweight CNNs, despite their efficiency and high internal consistency, must be complemented with robust domain-invariant learning mechanisms to achieve clinically reliable histopathological classification. Future work may focus on incorporating stain augmentation, transfer learning from large-scale pathology datasets, or self-supervised pretraining to enhance the resilience of the proposed framework against such domain shifts.

## Conclusion

This study presented a systematic exploration of lightweight convolutional neural network (CNN) architectures Lite-V0, Lite-V1, Lite-V2, and Lite-V4 for the automated classification of colon histopathological images. The proposed framework was designed to address the dual objectives of achieving high diagnostic accuracy and maintaining computational efficiency. A balanced dataset containing two primary classes, *Colon_Adenocarcinoma* and *Colon_Benign_Tissue*, was used for training, validation, and testing to simulate realistic clinical diagnostic workflows. The training process integrated advanced augmentation techniques, macro-F1-based early stopping, and extensive evaluation metrics including validation accuracy, loss, and F1 score, as well as confusion matrices, ROC, and precision-recall curves. Among all developed models, Lite-V2 demonstrated the most promising results, achieving a near-perfect validation macro-F1 score of 0.999 with an extremely compact model size of 1.53 MB and approximately one million parameters. This highlights the effectiveness of moderate-depth lightweight CNN architectures in maintaining high discriminative capacity while significantly reducing computational complexity. Such efficiency is crucial for deployment in clinical environments or on edge devices, where limited resources demand compact yet robust models. The results confirm that carefully optimized shallow models can match or even outperform deeper, resource-intensive networks in histopathological classification tasks.However, the test set evaluation revealed a clear challenge of domain shift, as the Lite-V2 model achieved only 50% accuracy and a macro-F1 score of 0.33 on unseen data. The model performed well on adenocarcinoma images but struggled with benign tissue recognition, suggesting that differences in slide staining, imaging quality, or dataset origin may have influenced generalization performance. This outcome emphasizes the need for robust domain adaptation and stain normalization methods in future work to ensure model reliability across varied data sources. The proposed framework provides an end-to-end reproducible training environment, employing a structured experimental design, balanced class weighting, and efficient model monitoring. It offers a foundation for developing practical, clinically applicable AI tools in digital pathology. The findings also underscore that model performance alone is insufficient; cross-domain generalization and interpretability remain critical for medical deployment.

## Data Availability

All data generated or analysed during this study are included in this published article^57^.
